# Opposing Effects of Neuronal Activity on Structural Plasticity

**DOI:** 10.3389/fnana.2016.00075

**Published:** 2016-06-28

**Authors:** Michael Fauth, Christian Tetzlaff

**Affiliations:** ^1^Department of Computational Neuroscience, Third Institute of Physics - Biophysics, Georg-August UniversityGöttingen, Germany; ^2^Bernstein Center for Computational NeuroscienceGöttingen, Germany; ^3^Max Planck Institute for Dynamics and Self-OrganizationGöttingen, Germany

**Keywords:** structural plasticity, architectural plasticity, timescales, synaptic plasticity, network topology

## Abstract

The connectivity of the brain is continuously adjusted to new environmental influences by several activity-dependent adaptive processes. The most investigated adaptive mechanism is activity-dependent functional or synaptic plasticity regulating the transmission efficacy of existing synapses. Another important but less prominently discussed adaptive process is structural plasticity, which changes the connectivity by the formation and deletion of synapses. In this review, we show, based on experimental evidence, that structural plasticity can be classified similar to synaptic plasticity into two categories: (i) Hebbian structural plasticity, which leads to an increase (decrease) of the number of synapses during phases of high (low) neuronal activity and (ii) homeostatic structural plasticity, which balances these changes by removing and adding synapses. Furthermore, based on experimental and theoretical insights, we argue that each type of structural plasticity fulfills a different function. While Hebbian structural changes enhance memory lifetime, storage capacity, and memory robustness, homeostatic structural plasticity self-organizes the connectivity of the neural network to assure stability. However, the link between functional synaptic and structural plasticity as well as the detailed interactions between Hebbian and homeostatic structural plasticity are more complex. This implies even richer dynamics requiring further experimental and theoretical investigations.

## Introduction

Information from the environment leads to the activation of neural subnetworks in the brain. The connectivity of these neural subnetworks, i.e., the existence and strength of synapses between neurons, influences the neuronal activation and, thereby, determines the way environmental information is processed. Accordingly, the long-term storage of information is related to activity-dependent (long-lasting) changes in connectivity (Hebb, 1949; Morris et al., 1986; Rioult-Pedotti et al., 1998; Leuner et al., 2003; Pastalkova et al., 2006; Whitlock et al., 2006; reviewed, e.g., in Martin et al., 2000; Chklovskii et al., 2004; Dudai, 2004; Hübener and Bonhoeffer, 2010). Basically two types of activity-dependent mechanisms yield such changes: synaptic or functional plasticity and structural plasticity. Structural or architectural plasticity determines the formation and removal of synapses. On the other hand, synaptic or functional plasticity changes the electrochemical transmission efficacy of synapses by altering, for instance, the receptor configuration of the postsynaptic site. Note, as we will show, this functional synaptic plasticity is associated with structural changes at existing synapses (size, postsynaptic density, etc.) and these changes are sometimes summarized as structural plasticity (Lamprecht and LeDoux, 2004). However, here we restrict structural plasticity to changes of the number of synapses (and of axonal/dendritic trees) and refer the long-term functional changes at existing synapses as synaptic plasticity.

The alterations of the transmission efficacy by synaptic plasticity depend on the level of neuronal activation. However, the mapping between activity level and triggered synaptic changes is not unique. In general, they are categorized into two classes: Hebbian and homeostatic synaptic plasticity. Hebbian synaptic plasticity yields an increase in synaptic efficacy given high neuronal activities (long-term potentiation; LTP; Bliss and Lomo, 1973; Lynch et al., 1983; Bliss and Collingridge, 1993; see Feldman, 2009 for a review), while low levels of activity induce a decrease (long-term depression; LTD; Lynch et al., 1977; Dudek and Bear, 1992; Mulkey and Malenka, 1992; see Collingridge et al., 2010 for a review). Thus, Hebbian synaptic plasticity basically maps the neuronal activation onto the synaptic efficacies or rather connectivity (high activity → stronger connections; low activity → weaker connections; Hebb, 1949; Bliss and Lomo, 1973; Dudek and Bear, 1992; Kirkwood et al., 1996). These changes in the connectivity, in turn, influence the neuronal activities. Along these lines, theoretical studies show (Rochester et al., 1956; Riedel and Schild, 1992; Gerstner and Kistler, 2002; Kolodziejski et al., 2010) that Hebbian synaptic plasticity alone induces a positive feedback loop leading to unrestricted synaptic (and thus neuronal) dynamics. On the other hand, homeostatic synaptic plasticity, as synaptic scaling (Turrigiano et al., 1998), act conversely to Hebbian synaptic plasticity. If neuronal activities are high, synaptic efficacies are decreased, while, if activities are low, efficacies are increased (high activity → weaker connections; low activity → stronger connections; Turrigiano et al., 1998; Hou et al., 2008, 2011; Ibata et al., 2008). Thereby, homeostatic synaptic plasticity alone induces a negative feedback loop and, thus, stabilizes the dynamics. As several theoretical results indicate (Tetzlaff et al., 2011; Zenke et al., 2013; Toyoizumi et al., 2014), the combination of both plasticity processes lead to desired, stable dynamics.

We will argue in this review that, analogous to functional synaptic plasticity, structural plasticity can also be categorized into two different classes of activity-dependency: (i) One class of structural changes maps features of the neuronal activity onto the connectivity, such that the connectivity is strengthened with high activity levels and vice versa. These changes will be referred to as Hebbian structural plasticity (Hebb, 1949; Helias et al., 2008). (ii) The other class of structural changes weakens (strengthens) the connectivity given high (low) neuronal activities and, thus, stabilizes the dynamics. This class is named homeostatic structural plasticity (Butz et al., 2009).

Note, this classification is phenomenological. Changes in connectivity (synaptic as well as structural) are not directly linked to neuronal activity. Neuronal activity initiates such changes by triggering secondary processes as molecular signaling cascades, which lead to the corresponding changes. For the here discussed plasticity processes, these underlying signaling cascades can have different degrees of similarity, which we will not consider in detail. The focus of this review is to systematize the qualitative links between the neuronal activity level and resulting connectivity changes.

Moreover, we focus on morphological changes of connections between excitatory neurons only. The dynamics of inhibitory synapses has been reviewed, for instance, by Vogels et al. (2013) for inhibitory synaptic plasticity and by Flores and Méndez (2014) for inhibitory structural plasticity. Further non-synaptic homeostatic mechanisms stabilizing neural network dynamics have been reviewed in Turrigiano and Nelson (2004), Marder and Goaillard (2006), or Yin and Yuan (2014).

In the following, as structural and synaptic plasticity are linked to each other, we first briefly outline the main findings for synaptic plasticity. Then, we review the morphological changes of synapses induced by synaptic plasticity and relate these changes to the dynamics of synapses and, thus, to structural plasticity. Following this, we summarize the experimental evidence of activity-dependent structural changes and categorize these, similar to synaptic plasticity, into the two classes of Hebbian and homeostatic structural plasticity. We also briefly review indications of Hebbian and homeostatic processes occurring during development. Finally, we sort theoretical investigations studying the dynamics of structural plasticity by this categorization and, based on their results, arrive at conclusions about the different functional roles of Hebbian and homeostatic structural plasticity.

## Activity-dependent synaptic plasticity

The most investigated long-term plasticity in neuronal systems is synaptic plasticity. This mechanism adapts synaptic efficacies (by, e.g., altering the number of AMPA receptors at the postsynaptic site) between neurons dependent on the neuronal activation. One distinguishes between two different forms of synaptic plasticity: (i) Hebbian synaptic plasticity and (ii) homeostatic synaptic plasticity.

- Hebbian synaptic plasticity adapts the synaptic efficacies seconds or minutes after onset of a stimulus-induced neuronal activation. In general, neuronal activity induces a calcium influx into the postsynaptic site inducing a complex molecular cascade which changes, amongst others, the number of AMPA receptors determining the synaptic efficacy (Kauer et al., 1988; Muller and Lynch, 1988; Shi et al., 1999; reviewed, e.g., in Malenka and Bear, 2004). Many experiments show that a low calcium level (thus a low neuronal activity level) leads to a decrease of the number of AMPA receptors (long-term depression: LTD; Lynch et al., 1977; Dudek and Bear, 1992; Mulkey and Malenka, 1992; Beattie et al., 2000; see Collingridge et al., 2010 for a review) while a high calcium level yields an insertion of new ones resulting in a stronger synaptic efficacy (long-term potentiation: LTP; Bliss and Lomo, 1973; Lynch et al., 1983; Malenka et al., 1992; Bliss and Collingridge, 1993; see Feldman, 2009 for a review). Thus, after several minutes, Hebbian synaptic plasticity maps the strength of the stimulus onto the strength of the synaptic transmission. Note, synapses from several input sources connecting to the same postsynaptic neuron can interact with each other yielding cooperative and competitive dynamics (Miller, 1996). Moreover, the change of the synaptic efficacy can also depend on the relative timing of pre- and postsynaptic action potentials (spike-timing-dependent plasticity: STDP; Levy and Steward, 1983; Gerstner et al., 1996; Markram et al., 1997b; Bi and Poo, 1998; see Markram et al., 2011 for a review), such that also temporal correlations might be mapped onto the synaptic efficacies. However, as several theoretical studies indicate, Hebbian synaptic plasticity alone induces a positive feedback loop leading to unrestricted growth of the synaptic efficacy (Rochester et al., 1956; Riedel and Schild, 1992; Gerstner and Kistler, 2002; Kolodziejski et al., 2010). In other words, if a stimulus drives the firing of the postsynaptic neuron, LTP potentiates the corresponding synaptic efficacy and, by this, induces a stronger input drive which, in turn, generates more potentiation and so forth. Thus, Hebbian synaptic plasticity alone would yield unstable, divergent dynamics of the synaptic efficacies.
- Another process adapting the transmission strength of a synapse is homeostatic synaptic plasticity. Several different homeostatic processes dampen the dynamics of neuronal systems on various levels (Zhang and Linden, 2003; Turrigiano and Nelson, 2004; Marder and Goaillard, 2006; Turrigiano, 2011; Yin and Yuan, 2014). Thus, it is reasonable that homeostatic processes, like synaptic scaling, also adapt synaptic efficacies (Turrigiano et al., 1998; Hengen et al., 2013; Keck et al., 2013). Amongst others, this mechanism depends mainly on the average postsynaptic activity (Ibata et al., 2008). Here, in contrast to Hebbian synaptic plasticity, if the neuronal activity is high, the synaptic efficacies are decreased and, if the activities are low, the efficacies are increased (Turrigiano et al., 1998; Burrone et al., 2002; Kim et al., 2012). Hereby, synaptic scaling is unspecific, i.e., it scales all synapses onto a postsynaptic neuron preserving relative differences between synaptic efficacies induced by Hebbian plasticity (Turrigiano, 2008). However, several experiments indicate (e.g., Turrigiano et al., 1998; Hengen et al., 2013; Keck et al., 2013, but see also Ibata et al., 2008) that, compared to Hebbian synaptic plasticity, this process is much slower (hours to days) which complicates the analysis of both processes within the same experimental setup (Vitureira and Goda, 2013). Nevertheless, theoretical investigations show that synaptic scaling is one way to solve the problem of unrestricted growth discussed above (Tetzlaff et al., 2011; Zenke et al., 2013; Toyoizumi et al., 2014). Please note that there are also other solutions proposed to solve this problem (von der Malsburg, 1973; Sejnowski, 1977a,b; Bienenstock et al., 1982; Oja, 1982).

In summary, investigations in the field of synaptic plasticity show that, at least, two classes of processes adapt synaptic efficacies: Hebbian synaptic plasticity and homeostatic synaptic plasticity. Hereby, Hebbian synaptic plasticity maps neuronal activities onto the synaptic efficacies (high act. → stronger connect.; low act. → weaker connect.), which are, in turn, stabilized by homeostatic processes (high act. → weaker connect.; low act. → stronger connect.).

## Activity-dependent structural plasticity

Activity-dependent structural plasticity basically influences two different physical substrates: On the one hand, *neurites* (i.e., dendrites and axons) grow and retract dependent on the level of neuronal activation (Cohan and Kater, 1986; van Huizen and Romijn, 1987). These growth processes determine the basic shape of a neuron and its regions of afferent and efferent connections. On the other hand, *synapses* (i.e., dendritic spines and axonal boutons) are continuously formed and deleted. Although an axon and a dendrite lie close together and the gap could be bridged by a synapse, the existence of a synapse is not guaranteed (Kalisman et al., 2005). In fact, the formation and deletion of a synapse also depend on the neuronal activation of both neurons (see e.g., Annis et al., 1994; Nägerl et al., 2007; Kwon and Sabatini, 2011; Hill and Zito, 2013).

As the majority of cortical synapses resides on dendritic spines (Yuste, 2010), many studies applied time-lapse imaging of the dynamics of dendritic spines for analyzing the structural dynamics or structural plasticity of single synapses. This implies the problem that the existence of a dendritic spine does not guarantee the existence of a functional synapse. However, several experiments provide evidence that, at least after a few hours after spine formation, new born spines are structurally and functionally equivalent to mature spines hosting a synapse (Trachtenberg et al., 2002; Knott et al., 2006; Nägerl et al., 2007; Zito et al., 2009). Similarly, also the emergence and stabilization of axonal terminals or boutons seems to involve synapse formation and maturation (Friedman et al., 2000; Ruthazer et al., 2006). Thus, the existence of a spine or bouton is a good indicator for the existence of a functional synapse.

### Link between structural and synaptic plasticity

The dynamics of synapses is determined by the dynamics of dendritic spines. Accordingly, structural plasticity depends on the morphology of spines as their sizes and shapes (Nägerl et al., 2008; Tønnesen et al., 2011, 2014). Experiments indicate that the volume of a dendritic spine correlates with the synaptic efficacy of the corresponding synapse (Matsuzaki et al., 2001; Knott et al., 2006; Zito et al., 2009) which, in turn, is influenced by synaptic plasticity. Accordingly, stimuli causing long-term potentiation (LTP) also cause spine enlargements (Fifková and Van Harreveld, 1977; Okamoto et al., 2004; Yang et al., 2008, for a review see Yuste and Bonhoeffer, 2001) while stimuli causing long-term depression (LTD) induce spine shrinkage (Okamoto et al., 2004; Zhou et al., 2004; Oh et al., 2013). Hereby, synaptic and structural changes rely on distinct signaling cascades, which are triggered by the same signals (Matsuzaki et al., 2004; Zhou et al., 2004). Thus, blocking synaptic plasticity, for instance, by blocking NMDA-receptors also prevents changes in the spine volume. Several experiments indicate that the spine head volume is correlated to the lifetime or stability of the spine (Grutzendler et al., 2002; Majewska et al., 2006; Yasumatsu et al., 2008; Loewenstein et al., 2015). Thus, the spine stability or removal of a synapse is, in turn, correlated to the synaptic efficacy of the corresponding synapse, which also has been directly observed in several experiments (Holtmaat et al., 2005; Le Bé and Markram, 2006, reviewed, e.g., in Kasai et al., 2003). In combination with STDP, this relation between synaptic weight, spine volume and spine stability could give rise to a spike-timing-dependent structural plasticity (Helias et al., 2008; Deger et al., 2012), which still has to be experimentally verified. Interestingly, the stability of a synapse is also influenced by the reliability of signal transmission of the synapse (Wiegert and Oertner, 2013) which is also altered by synaptic plasticity (Stevens and Wang, 1994). Thus, for Hebbian-like changes, structural and synaptic plasticity are linked with each other by the morphology of spines or properties of the synapse (Segal, 2005).

Some evidence indicates a similar link for homeostatic changes: *in vitro* (Murthy et al., 2001) and *in vivo* (Keck et al., 2013) studies show that changes of the spine volume also go along with the activity-dependent homeostatic scaling of synaptic efficacies. Given the aforementioned correlation between spine volume and spine stability, we expect that structural plasticity is also linked to homeostatic synaptic plasticity.

In the following, we will summarize experimental results indicating the different aspects of activity-dependent structural plasticity in more detail. We will classify these aspects according to Hebbian (high act. → stronger connect.; low act. → weaker connect.) and homeostatic (high act. → weaker connect.; low act. → stronger connect.) structural plasticity in adult networks. In addition, we will show that many of these experiments support the here discussed link between synaptic and structural plasticity. We will also provide a brief survey of structural dynamics during development. Finally, we will discuss experimental evidence of the interaction of Hebbian and homeostatic structural plasticity in the same neural system.

### Evidence for hebbian structural plasticity

#### LTP-stimuli

The induction of LTP by a strong neuronal activation is mainly associated with the increase of the synaptic efficacy (e.g., number of AMPA receptors) of existing synapses (Malenka and Bear, 2004; Feldman, 2009). However, already in the 1980s first studies (Lee et al., 1980; Chang and Greenough, 1984) indicate that 15–20 min after applying the strong stimulus the number of synapses is enhanced, too. In addition, one also observes an increase in the number of filopodia (Lee et al., 1980; Chang and Greenough, 1984), which seem to be the precursors of dendritic spines (Ziv and Smith, 1996). Accordingly, about 30 min after stimulation, an increased number of dendritic spines can be observed (Moser et al., 1994; Trommald et al., 1996; Engert and Bonhoeffer, 1999, but see also Desmond and Levy, 1990). The strength of the effect and the detailed timescale, however, depend strongly on the used tissue and preparation method (Sorra and Harris, 1998; Dunaevsky et al., 1999; Kirov et al., 1999; Bourne et al., 2007; Bourne and Harris, 2011), but most studies report timescales between 5 and 30 min.

This increase in the number of spines after an LTP-stimulus provides further support for an interaction between Hebbian synaptic plasticity and Hebbian structural plasticity: A strong neuronal activation will induce an increase in synaptic efficacies or rather in the spine volumes implying the stabilization of these enlarged dendritic spines. Given a continuous formation of new spines, this also implies that the new and small spines, which would be pruned without stimulation, will be enlarged and stabilized by synaptic plasticity. Together with the already existing (and further stabilized) spines, the stabilization of new spines by the strong stimulus would lead to an increase of the the number of spines as observed experimentally. For this, the rate of forming new spines could be independent of the neuronal activity and stay constant. This potential explanation of the increase in spine number is supported by a recent study demonstrating that LTP stabilizes nascent spines (Hill and Zito, 2013). Accordingly, blocking the signals inducing LTP (by blocking the NMDA-channels) also prevents the increase in the number of dendritic spines and also of axonal boutons (Engert and Bonhoeffer, 1999; Maletic-Savatic et al., 1999; Toni et al., 1999; Nikonenko et al., 2003). Thus, the dynamics of dendritic spines can be explained by the link between Hebbian structural plasticity and synaptic plasticity.

Note, although an increase of the number of spines could be explained by assuming a constant rate of forming new spines, the LTP-dependent appearance of more filopodia (Lee et al., 1980; Chang and Greenough, 1984; Maletic-Savatic et al., 1999) suggests that the formation rate changes, too. Thus, further experiments are required to clarify whether the formation rate of dendritic spines (and also of axonal boutons) stays constant or whether it is adapted by the level of neuronal activity.

Also at the presynaptic neuron an LTP-inducing stimulus triggers a structural remodeling: the number of axonal boutons increases. This effect arises already 15 min after the stimulation (Nikonenko et al., 2003; Ninan et al., 2006). The fact, that both the numbers of dendritic spines and of axonal boutons are enhanced, suggests that new synapses are formed by these new elements. In addition, recent findings indicate also an LTP-dependent increase in the probability that a bouton hosts one or more functional synapses (Medvedev et al., 2014). Thus, newly formed spines have a very high chance of connecting to a new or old bouton and, hence, forming a new synapse.

#### LTD-stimuli

A link between the dynamics of Hebbian structural plasticity and LTD-inducing stimuli has been established, too. Several experimental studies show that the induction of an LTD-stimulus (low frequency) yields a separation of pre- and postsynaptic terminals (Bastrikova et al., 2008) and a loss of dendritic spines (Nägerl et al., 2004; Wiegert and Oertner, 2013). Thus, similar to the dynamics triggered by an LTP-stimulus, due to the induction of a low frequency stimulation, the synaptic efficacy is decreased, spines shrink and decrease their stability, and the removal rates of dendritic spines are increased (Segal, 2005). This is supported by experiments showing that the prevention of LTD by blocking NMDA-channels impedes the structural effects (Nägerl et al., 2004; Yu et al., 2013). Thus, also these results indicate that structural plasticity is linked to synaptic plasticity which influences the stability of the corresponding dendritic spines. The timescale of spine shrinkage and removal seems to depend on the experimental conditions: some experiments report spine shrinkage after about 20 min of LTD-induction (Oh et al., 2013) while other studies report no significant changes in spine volume or stability up to 30 min after the induction of LTD (Wiegert and Oertner, 2013). Like for LTP-induced dynamics, also the presynaptic site is also influenced by a low level of activation as it increases the turnover of axonal boutons (Paola et al., 2006; Stettler et al., 2006) resulting in a loss of synapses (Becker et al., 2008).

In summary, stimulation protocols inducing Hebbian synaptic plasticity change the stability and number of synapses. A strong activation induces the formation of more synapses while a low activation induces a loss of synapses. These variations in the number of synapses seem to depend on changes in the stability of the corresponding dendritic spines and axonal boutons correlated to the actual synaptic efficacy adapted by Hebbian synaptic plasticity. However, it is still not clear whether the rate of newly formed spines and boutons is changed, too. Furthermore, the data about the dynamics induced by LTD-stimuli are less comprehensive than the data for LTP-stimuli.

Most of the above discussed structural dynamics happens on a timescale of the order of several minutes to one hour. On this timescale the dendritic trees and axons hosting spines and boutons remain quite stable (Ziv and Smith, 1996; Grutzendler et al., 2002; Trachtenberg et al., 2002; Lee et al., 2006; Paola et al., 2006; Stettler et al., 2006). Hence, fast Hebbian changes of the network structure must be mainly implemented by the growth or removal of dendritic spines. On slower timescales, also changes of the dendrites and axons take place. However, as we will discuss in the following, such changes are mainly triggered by homeostatic processes.

### Evidence for homeostatic plasticity

As already mentioned above, the connectivity of neural networks is not only adapted by Hebbian-like changes. Similar to synaptic plasticity, also structural changes show homeostatic dynamics, i.e., a decrease of connectivity with high neuronal activities and an increase with low activities. Typically, these homeostatic dynamics are observed under chronically altered conditions of neuronal activity and, thus, also at slower timescales. In general, the resulting structural changes seem to counterbalance the altered conditions and, thereby, regulate the activity back to an intermediate level (for a complete review of homeostatic structural processes see Butz et al., 2009). Like Hebbian structural plasticity, homeostatic structural changes are determined by the dynamics of dendritic spines and axonal boutons. However, under extreme conditions, as in epilepsy or after lesions, also changes of the dendritic and axonal trees are observed.

Already in the year 1978, Wolff et al. (1978) observed *in vivo* the growth of protrusions and thickenings on the dendritic tree after decreasing neuronal activity. For this, they applied the inhibitory neurotransmitter GABA for 3–7 days. Further studies verified that chronic blockage of neuronal activity can yield an increase in the number of spines after approximately 8 h (Dalva et al., 1994; Rocha and Sur, 1995; McAllister et al., 1996; Kirov and Harris, 1999) indicating a slower timescale for homeostatic structural changes as compared to Hebbian ones. Hereby, already the blockage of NMDA channels leads to an increase in spine number (Yu et al., 2013; Chen et al., 2015) or prevents spine elimination (Bock and Braun, 1999). Note that during development blocking activity or NMDA receptors can show the opposite effect (Annis et al., 1994; Collin et al., 1997). However, the newly formed spines often host silent synapses needing synaptic plasticity to be converted to functional synapses (Nakayama et al., 2005). On the other hand, persistent depolarization of neurons leads to a loss of dendritic spines (Müller et al., 1993; Drakew et al., 1996). Already the application of high levels of NMDA induces a spine loss by the destabilization of the spine actin scaffold (Halpain et al., 1998). Thus, the number of spines is adapted in an activity-dependent homeostatic manner.

Furthermore, the changes in the number of spines also depend on the calcium level (Kirov and Harris, 1999; Kirov et al., 2004; Tian et al., 2010). Accordingly, it has been proposed that dendritic spines follow a calcium-dependent homeostasis (Segal et al., 2000). As the postsynaptic calcium level is largely influenced by neuronal activity (Spruston et al., 1995; Helmchen et al., 1996; reviewed, e.g., in Higley and Sabatini, 2008), the calcium-dependent homeostasis could, in turn, imply an activity-dependent homeostasis as described above. However, the detailed relation between calcium, activity, and spine dynamics is more complex, as the calcium level is also regulated by other signals as neurotrophins (Stoop and Poo, 1996) or cell adhesion molecules (Bixby et al., 1994). Furthermore, in contrast to the postsynaptic activity, calcium is a local signal allowing different dynamics at different branches of the dendritic tree. Accordingly, by comparing different branches of the same dendrite, where each branch receives stimuli from other brain regions, such different spine dynamics are observed (Mattson, 1988; Bravin et al., 1999; Lohmann et al., 2005; Deller et al., 2006; Vuksic et al., 2011, see also Yu and Goda, 2009; Vlachos et al., 2012a, 2013 for evidence on local homeostasis of synaptic efficacies). However, in summary, these experiments indicate that the number of spines or synapses is adapted by activity-dependent homeostatic structural plasticity.

#### Evidence from networks in extreme situations

Further evidence for homeostatic dynamics are obtained in more complex settings which we summarize in the following. Note that under these conditions dynamics of dendritic and axonal trees are observed, too.

For example, homeostatic regulation of connectivity is found in animal models of epilepsy. Epileptic seizures are network states of high and synchronous activity. Given a homeostatic dynamic, this would lead to a decrease in the number of spines which, indeed, was found in animal models of epilepsy (Scheibel et al., 1974; Paul and Scheibel, 1986; Geinisman et al., 1990; Isokawa and Levesque, 1991; Isokawa, 1998). These changes are likely signs of structural plasticity rather than mere damages by the epileptic seizures, as the number of spines recovers after several days without seizures (Müller et al., 1993; Isokawa, 1998). The spine loss is only visible at least 5 h after the seizure (Mizrahi et al., 2004), which implies that, also under these conditions, the timescale of homeostatic structural plasticity is typically slower than for Hebbian structural plasticity described above. Interestingly, after several days with reoccurring seizures also changes of the neuronal morphology, like retraction of dendritic branches, are measured (Colling et al., 1996; Jiang et al., 1998).

In contrast to the elevated activities during epilepsy, phenomena like strokes, lesions, or deprivations typically lead to lowered activity levels in a group of neurons. For instance, for deprived neurons a homeostatic dynamic would increase the number of spines. Indeed, experiments show that, after 4 days of monocular deprivation, the number of newly formed spines in the binocular cortex of adult mice doubles compared to control conditions (Hofer et al., 2009). Interestingly, a second phase of monocular deprivation at the same eye does not lead to an increased formation of new spines. Now, the synaptic efficacies of spines formed during the first phase are strengthened by (presumably homeostatic) synaptic plasticity counterbalancing the lost input (Hofer et al., 2009). These findings support the link between (homeostatic) structural and synaptic plasticity.

However, as shown by Keck et al. (2008), also smaller interferences, like small lesions of the retina, lead to more new spines (in the lesion projection zone in the visual cortex). In this experiment, although more new spines are formed, the spine density is comparable to control conditions after 3 days. Another experiment shows that trimming the whiskers of rats leads to an increased number of spines and an outgrowth of dendritic trees in the input-receiving layer in the barrel cortex (Vees et al., 1998; other layers might be affected differently, see Chen et al., 2015). Along this line, one observes massive reorientation of the dendritic trees of adult rats after whisker removal, while the system regains the pre-removal dendritic lengths and spine densities (Tailby et al., 2005). Note, however, already the retraction of dendrites from denervated areas can increase the exitability of neurons, such that activity-homeostasis can be reached without regaining the pre-lesion dendritic length (Platschek et al., 2016).

Interestingly, not only the dendrites of the neurons with lesioned afferents, but also axons of neighboring neurons contribute to regain homeostasis. Although these neighboring neurons are not directly affected by the lesions, they can also be expected to experience altered activity levels. This triggers, after a few days, the growth of axons from the neighboring neurons toward the deprived region (Darian-Smith and Gilbert, 1994; Yamahachi et al., 2009; Marik et al., 2010). Furthermore, damaged axons can grow out and form new synapses, similar to growth dynamics during development (Canty et al., 2013). In summary, we find that lesions trigger the formation of new spines and the outgrowth of dendrites, which, together with new innervation from neighboring neurons, presumably form new synapses and restore the activity level.

Thus, very high or low activity levels occurring in extreme situations like epilepsy, lesions, or stroke are counterbalanced by structural changes on the timescale of several hours to days, thereby, contributing to activity-dependent homeostasis.

### Activity-dependent structural plasticity during development

As already mentioned above, apart from networks in extreme situations, many experiments in adult networks observe very small or no changes of the axonal or dendritic arborization. This is different during the development of neural networks, when these dendritic and axonal trees are formed. Interestingly, also during this phase activity-dependent structural processes contribute to the network dynamics. In the following we will briefly discuss these experiments.

#### Homeostatic structural changes during development

Single, isolated neurons in culture typically start growing axons and dendrites. This initial process could already be a homeostatic mechanism, as such neurons typically exhibit only weak activities (Kater et al., 1989). The further outgrowth of neurites also seems to be homeostatically regulated: On the one hand, the application of the inhibitory neurotransmitter GABA, which normally decreases activity, triggers an increased outgrowth (Mattson and Kater, 1989). On the other hand, excitatory neurotransmitters as glutamate (but not NMDA, see Mattson et al., 1988), which normally yield an increased activity, induce the degeneration of the dendritic structures (Haydon et al., 1984, 1987; Mattson, 1988; Mattson et al., 1988; Mattson and Kater, 1989). The strength of this effect is dose-dependent (Mattson et al., 1988). Note, during early developmental phases, GABA is an excitatory neurotransmitter (Barker et al., 1998; Ben-Ari, 2002). Still, in the above studies GABA shows the inverse effect of the excitatory neurotransmitters. Furthermore, in these experiments, changes in the axonal dynamics are initiated only at very high doses and lead to a retraction of the axon.

Further experiments targeted downstream signals of these neurotransmitters. Also here, several indications show that especially the postsynaptic calcium level seems to trigger dendritic changes: on a slower timescale, an increased level of calcium induces a retraction of dendrites while a decrease of calcium leads to an outgrowth of dendrites (Mattson and Kater, 1987, for a similar effect for CaMKII see Wu and Cline, 1998). These dynamics are summarized in the calcium-dependent homeostasis hypothesis for dendrites (Kater et al., 1989; Lipton and Kater, 1989). Furthermore, recent experiment suggest that also the dynamics of filopodia are regulated dependent on local calcium currents (Lohmann et al., 2005). As discussed above, the calcium level is mainly influenced by the neuronal activity. Therefore, we suppose that the calcium-dependent homeostasis hypothesis implies an activity-dependent homeostasis, i.e., neurons grow and retract their dendrites to find an optimal level of input which, in turn, assures a medium activity level. This hypothesis is supported by experiments showing that increased activity, due to electrical stimulation, prevents dendritic outgrowth (Cohan and Kater, 1986; Fields et al., 1990 but see Garyantes and Regehr, 1992), whereas blocking activity yields enhanced growth of dendrites (van Huizen and Romijn, 1987; Fields et al., 1990). Note, these experiments demonstrate a relation between activity and the dendritic outgrowth describing only the potential connectivity between neurons and not the realized connectivity between neurons. Whether this also yields the formation of more functional synapses remains unclear.

Further evidence for homeostatic structural changes during development are coming from experiments analyzing the time course of the developmental process of neuronal networks. During development, neural networks evolve from an initial unconnected state to a connected matured state. Initially, neurons have very low activities (Ramakers et al., 1990; Chiappalone et al., 2006; Wagenaar et al., 2006), which could trigger the outgrowth of neurites and formation of synapses in a homeostatic manner (van Ooyen, 2011). Before reaching the matured state, neural networks typically pass through a phase of extreme build up of synapses followed by a phase of synapse pruning (so-called overshoot)—dependent on the level of neuronal activity (Feldman and Dowd, 1975; Huttenlocher et al., 1982; Huttenlocher, 1984; van Huizen et al., 1985, 1987; van Huizen and Romijn, 1987; van Pelt et al., 1996; Bock and Braun, 1999; Hua and Smith, 2004; Zuo et al., 2005a,b). Such an overshoot in synapse number is typical for neural networks with homeostatically regulated connectivity (van Ooyen, 2003, 2011).

#### Hebbian structural changes during development

During development some structural changes of axonal and dendritic trees also show Hebbian-like dynamics as described in the following: Neurites grow by constantly adding and removing branches (Wu and Cline, 1998; Sin et al., 2002; Wong and Ghosh, 2002; Portera-Cailliau et al., 2003). Hereby, only a few branches become stable and form the axonal or dendritic tree, while others are removed on the timescale of minutes to hours (Wu and Cline, 1998). Thereby, the activation of receptors and local calcium transients are necessary to stabilize and maintain dendritic branches (Lohmann et al., 2002; Vaillant et al., 2002; Hutchins and Kalil, 2008). Accordingly, in animals experiencing four hours of increased neuronal activity due to visual stimulation, one observes significantly more stabilized dendritic branches as compared to animals left in the dark (Sin et al., 2002). Similarly, the blockage of neuronal activities yields much less complex dendritic trees (Groc et al., 2002).

Interestingly, the stabilization of dendritic and axonal branches also depends on the connectivity, more precisely, on the existence and maturation of synaptic contacts on the branch (Haas et al., 2006; Ruthazer et al., 2006). As shown above, in adult networks, the activity-stability relationship of synapses implements Hebbian changes in connectivity. Thus, if the dynamics underlying the stabilization of synaptic contacts are similar during development and in adult networks, the activity-dependent stabilization of spines and, therefore, of branches would indicate a Hebbian-component of the growth of dendritic trees.

## Evidence for the interaction of hebbian and homeostatic structural plasticity

**Figure 1:**
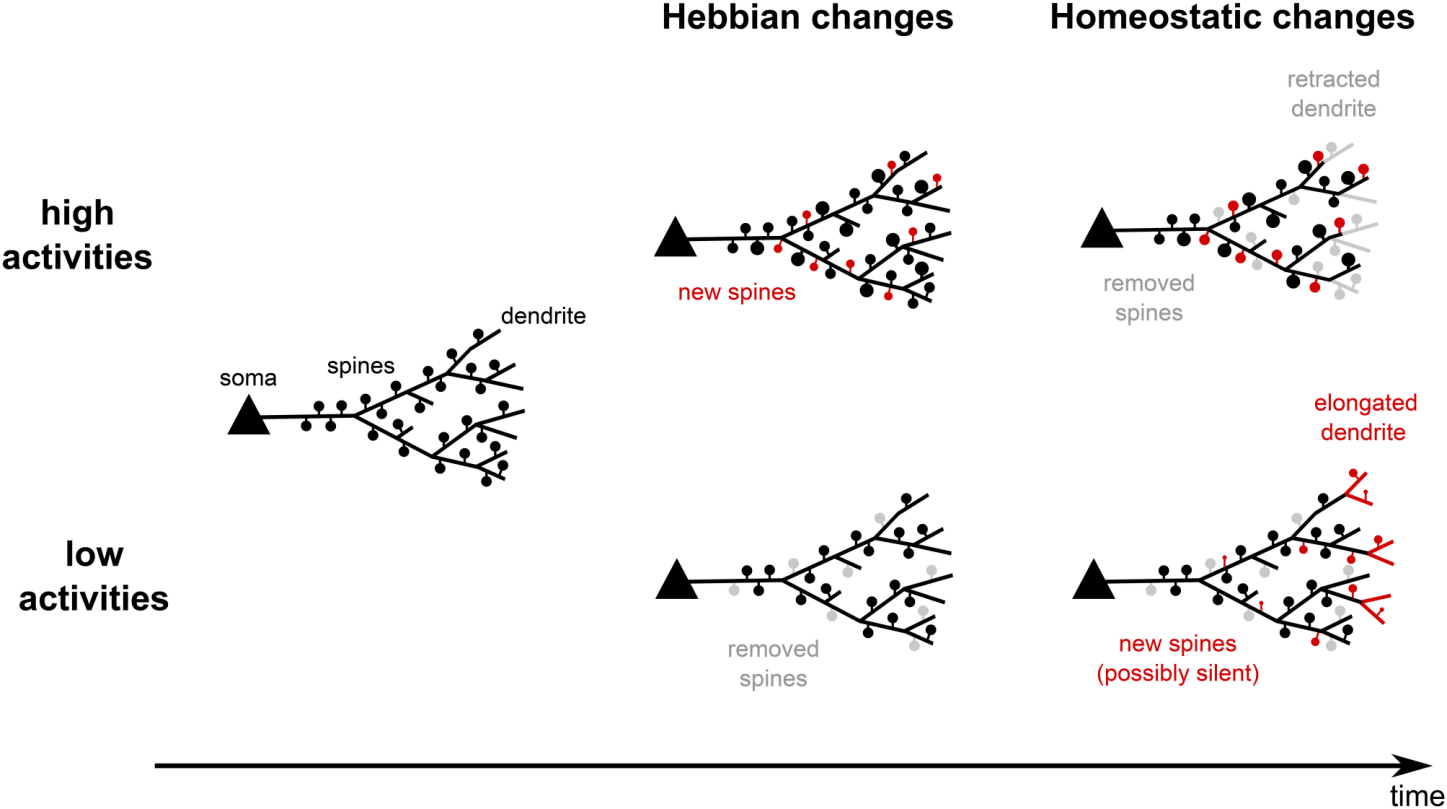
**Summary of experimental findings**. On the one hand, neural systems, which experience high activities, quickly form new spines and strengthen old ones (Hebbian changes). If high activities persist for longer time, spines are removed and dendrites start to retract. This reduces the input and drives the postsynaptic activities back to lower levels (homeostatic changes). On the other hand, low activities lead to spine removal and shrinkage. On the long run, however, new spines are created and dendrites start growing out such that the neurons acquire more inputs and increase their activity levels. Thus, structural plasticity shows Hebbian-type changes and homeostatic changes.

As we discussed above, for adult networks, the alteration in neuronal activity causes two different directions of structural changes (see Figure 1). On a fast timescale (minutes to hours) the number of dendritic spines goes along with the change in neuronal activity in a Hebbian manner. On a typically slower timescale (hours to days), the dynamics of dendrites and dendritic spines homeostatically counterbalance the change in activity and regulate it back to an intermediate target regime. Obviously, in experiments, chronic changes in neuronal activity should trigger both processes which, then, interact with each other. With these two mechanisms and their typically different timescales at hand, in the following, we will discuss direct conclusions about the dynamics of structural changes during a period of altered activity.

For example, when neurons start to receive reduced or LTD-inducing inputs, the corresponding synapses will be depressed and, therefore, more likely to be removed due to Hebbian structural plasticity—the spine density is reduced (Figure 1, bottom center). Later on, due to the reduced activity of the neuron, homeostatic structural plasticity yields the formation of new synapses—the spine density will increase (Figure 1, bottom right). Note, as the homeostatic changes are unspecific, very likely these new synapses connect to other, more active inputs. Thus, when the neural network has again reached its homeostatic level and assuming that the synaptic efficacies are, on the long run, similar to those before the activity alteration, the spine density is probably at the same level as before receiving the LTD-inducing inputs. Thus, as a direct consequence from the interaction of Hebbian and homeostatic structural plasticity in the same neural network, we expect in general a transient decrease in the spine density.

Such transient changes have been observed already in the 1970s (Parnavelas et al., 1974; Goldowitz et al., 1979). In these studies, the transsection of afferent hippocampal axons yields a strong decrease in spine density around 4 days after deafferentiation and a restoration of the initial spine density after 10–50 days (Parnavelas et al., 1974; Goldowitz et al., 1979; Vuksic et al., 2011). Strikingly, this transient change in spine density does not result from changes in the spine formation rate, but rather from changing the elimination rate or the stability of the spines (Vlachos et al., 2012b). Similarly, one observes changes in the spine elimination rate in barrel cortex after whisker trimming also leading to a transient decrease of the spine density (Zuo et al., 2005a; Miquelajauregui et al., 2015).

These results are consistent with the correlation between spine stability, spine volume, and synaptic efficacy governing the interaction of synaptic and structural plasticity: First, Hebbian synaptic plasticity would decrease the efficacies and the stability of spines, such that their density decreases. Later, synaptic scaling would scale up the synaptic efficacies of both old and new synapses and, thereby, stabilizes them and increases spine density. Interestingly, at the same time, Hebbian synaptic plasticity can induce competitive effects between newly formed and up-scaled preexisting spines, which destabilizes the newly formed synapses and, thereby, protracts the recovery of the system (Vlachos et al., 2013).

On the other side, paradigms which supposedly trigger higher neural activities, such as motor learning or an enriched environment, have been demonstrated to elicit a transient increase in the number of spines after 2–3 days of stimulation. After 7 days the number of spines reaches control level again (Xu et al., 2009; Yang et al., 2009, see Figure 1, upper row). Also during these experiments, the number of new filopodia remains constant, which suggest a constant formation rate of new spines. Interestingly, the repeated training selectively stabilizes mainly the newly formed spines, while the stability of preexisting spines drops (Xu et al., 2009). Also for these types of experiments the interaction of structural and synaptic plasticity provides a potential explanation for the observed dynamics. We expect that Hebbian synaptic plasticity leads to a selective potentiation and, thus, a stabilization of the synapses which are important for learning (especially the ones hosted by newly formed spines, which are important for the task performance, see Xu et al., 2009; Yang et al., 2009). This, in turn, leads to an increased spine number and higher neuronal activities. In the long run, this increased activity triggers unspecific homeostatic synaptic plasticity decreasing the stability of synapses and inducing their pruning. Remarkably, in experiments, when training is stopped earlier, the newly formed spines are less stable than the preexisting ones (Xu et al., 2009). Following our reasoning, this could imply that learning was not long enough to trigger sufficient potentiation to stabilize the newly formed synapses.

The interaction of Hebbian and homeostatic mechanisms could also be used to explain a detailed EM-study conducted by Bourne and Harris (2011). This study shows that, 5–30 min after a typically LTP-inducing tetanic burst stimulation, a transient increase in the number of stubby spines, shaft synapses, and nonsynaptic protrusions can be observed. However, already after 2 h these structures are not present anymore. In addition, the number of small spines is decreased compared to pre-stimulation, whereas the postsynaptic densities of all remaining spines have been enlarged such that the PSD (postsynaptic density) area per micrometer dendrite is the same as for controls (Bourne and Harris, 2011). This suggests a strong and, possibly, fast homeostatic mechanism (the authors argue for a resource homeostasis of the polyribosomes which are used for spine creation and enlargement). Thus, probably a group of synapses is selectively stabilized by Hebbian synaptic plasticity increasing neuronal activity. At the same time, homeostatic synaptic and structural plasticity counterbalance these changes and decrease the stability of all synapses leading to the removal of small, unpotentiated synapses. These dynamics are similar to the dynamics during motor learning described above.

These examples demonstrate that Hebbian and homeostatic as well as synaptic and structural plasticity are strongly interweaved and jointly adapt the connectivity of the neural network according to alterations in neuronal activity. To understand these complex interactions in more detail, further experiments are needed. However, to assess also the general principles, theoretical network models are required. In the following, we will discuss the state of the art of theoretical models of structural plasticity.

## Theoretical models of structural plasticity

In this section, we will summarize theoretical and computational studies analyzing the dynamics and functional consequences of structural plasticity. As models of structural plasticity basically adapt the connectivity, they enable predictions about properties of the connectivity in neural networks. These properties range from statistical (e.g., the statistics of subnetwork structures (motifs) or the probability distribution of the number of synapses between two neurons) to graph theoretical features (e.g., small-worldness or shortest path lengths) which can be compared to biological data. Many studies also investigate functional consequences of structural plasticity as, for instance, the influence on the storage capacity or the ability to classify different inputs. The majority of studies focuses on either Hebbian or homeostatic structural plasticity, however, at the end of this section, we will provide an overview of the few studies combining both processes of structural plasticity.

### Hebbian structural plasticity

As discussed above, Hebbian structural plasticity is mainly realized by the dynamics of dendritic spines. Thus, models of Hebbian structural plasticity typically describe the dynamics of dendritic spines. Synapses in these models appear and disappear at predefined potential synaptic locations with certain probabilities influenced by neuronal activities, synaptic efficacies and/or other hidden variables. As activities and efficacies depend on synaptic plasticity, Hebbian structural plasticity and Hebbian synaptic plasticity are strongly interconnected and the majority of models of Hebbian structural plasticity also incorporate the dynamics of Hebbian synaptic plasticity and some even homeostatic synaptic plasticity.

The simplest neural network to study the influence of Hebbian structural plasticity on the network's dynamics and connectivity is a postsynaptic neuron receiving input from one presynaptic neuron. Several experiments show that the connectivity between such pairs of neurons (the probability distribution of the number of synapses) is non-trivial (Markram et al., 1997a; Feldmeyer et al., 1999, 2002, 2006; Hardingham et al., 2010): these neurons are either unconnected (no synapse) or connected by *multiple* synapses (four to five synapses). This finding does not depend on the detailed anatomy of neurons, as the number of potential synapse location is much higher than the number of realized synapses (Fares and Stepanyants, 2009). However, as theoretical models show (Deger et al., 2012; Fauth et al., 2015b), Hebbian structural plasticity yields the formation of such multi-synaptic connections in a broad range of activity levels. By changing the activity level, the number of synapses between the neurons can be adjusted providing a way to change connectivity and, thus, store information in an activity-dependent manner (Fauth et al., 2015a,b). Furthermore, although the storage capacity per synapse is decreased, information, stored in such structures, can persist for timescales much longer than the lifetime of a single synapse, as the storage is collectively implemented by all synapses and does not rely on the existence of single ones (Fauth et al., 2015a).

Instead of considering a system consisting of one postsynaptic neuron, which receives inputs from one presynaptic neuron by multiple synapses, other studies considered a slightly more complex system: a postsynaptic neuron receiving inputs from several presynaptic neurons (note that in this system each presynaptic neuron is considered to be connected by only one synapse to the postsynaptic neuron). Here, the stability of synapses depends on the activity-dependent calcium influx; a high calcium influx causes stabilization of synapses and a low influx implies destabilization of synapses. Similar, as for the multi-synaptic connections, high neuronal activities lead to a stabilization of all synapses (Helias et al., 2008). However, for intermediate activity levels only correlated inputs are stabilized. Thus, the information stored in the connectivity could also be the information about the correlations between different inputs (Helias et al., 2008). In addition, synapses from uncorrelated inputs are pruned or deleted and lose their (noisy) influence on the postsynaptic neuron (Helias et al., 2008). Thus, Hebbian structural plasticity might help to prune synapses which are unimportant for the dynamics of the neural network.

Accordingly, also in more complex and biologically more reasonable systems, as large recurrent networks (Bourjaily and Miller, 2011; Zheng et al., 2013; Miner and Triesch, 2016), synaptic pruning preferentially removes synapses which only weakly contribute to synaptic transmission. These models use synaptic plasticity rules which typically yield a bimodal distribution of the electrical transmission efficacies with many efficacies close to zero. In combination with synaptic pruning, however, synapses with small efficacies are removed leading to the emergence of a unimodal distribution as observed in the cortex (Song et al., 2005). Accordingly, the continuous pruning and creation of synapses can also be interpreted as a process of stochastic inference, in which the network continuously tests and evaluates the “usefulness” of synapses to process or represent external stimuli (Kappel et al., 2015). Thus, synaptic pruning might minimize the resources for synaptic maintenance while preserving important dynamics.

Further advantages of pruning or deletion of uncorrelated or unimportant synapses have been revealed for simpler feedforward neuronal networks, which are typically used to study associative memory: the storage capacity of these networks is increased (Knoblauch et al., 2009, 2014). Considering a Willshaw or Hopfield network (Willshaw et al., 1969; Hopfield, 1982), the deletion of the weak or unimportant synapses increases the storage capacity per synapse without perturbing the stored patterns (Knoblauch et al., 2009). Furthermore, pruning prevents the occurrence of catastrophic forgetting and could explain phenomena as retrograde amnesia or the difference between spaced- and block-learning (Knoblauch, 2009; Knoblauch et al., 2014). Intuitively, the increase in storage capacity per synapse contradicts the finding of multi-synaptic connections described above (Deger et al., 2012; Fauth et al., 2015b). However, the influence of multi-synaptic connections on memory has to be further investigated as models at the network level are so far missing.

So far, theoretical studies of structural plasticity in recurrent networks mostly investigated storage capacity and compared the properties of the resulting connectivity with the properties of biological measured connectivities as, for instance, the statistics of the so-called motifs (Milo et al., 2002), i.e., configurations of the connectivity in small subnetworks. In cortical networks, groups of strongly connected neurons show an increased appearance (compared to random networks; Markram et al., 1997a; Feldmeyer et al., 1999; Song et al., 2005; Perin et al., 2011). As groups of strongly connected neurons typically show strongly correlated activities, which, in turn lead to stabilization of the corresponding connections, this increased appearance is naturally reproduced by Hebbian structural plasticity interacting with synaptic plasticity (Bourjaily and Miller, 2011; Miner and Triesch, 2016, but see also Zheng and Triesch, 2014). Remarkably, with the formation of more strongly connected subgroups of neurons the network's performance in discriminating different inputs increases (Bourjaily and Miller, 2011).

In summary, these results show that Hebbian structural plasticity improves several properties of neural networks compared to networks adapted only by synaptic plasticity. Especially, the storage of memories is improved in storage lifetime, capacity, and noise robustness. Furthermore, perhaps related to these improvements in memory storage, also the ability to discriminate inputs is enhanced. However, further investigations are needed to understand the influence of Hebbian structural plasticity on the dynamics of neural networks.

### Homeostatic structural plasticity

As already described above, homeostatic structural plasticity adapts dendrites and axons dependent on the neuronal activity to reach and sustain an intermediate activity regime (Butz et al., 2009). The slow timescale of homeostatic structural plasticity implies that its influences are basically observed after long durations, as during development, or in networks under extreme activity conditions as after lesions. Thus, also theoretical models investigating the dynamics of homeostatic structural plasticity concentrate mainly on these two paradigms.

During the development of a neural network from a naive initial state to a matured network, it passes through an overshoot phase of building up many synapses followed by a pruning phase until the network settles in the ground state (van Huizen et al., 1985, 1987; van Huizen and Romijn, 1987; van Ooyen, 2003, 2011). Such dynamics are already seen in a pure excitatory network model governed by homeostatic structural plasticity without the differentiation between axons and dendrites (van Ooyen and van Pelt, 1994). Introducing also inhibition further pronounces this overshoot effect and can lead to oscillatory and bursting neuronal activities (van Ooyen et al., 1995; van Ooyen and van Pelt, 1996). Assuming different homeostatic dynamics for axons and dendrites results in even more complex activity dynamics matching cell culture data (Tetzlaff et al., 2010). The resulting network state is the so-called critical state which is predestined for maintaining stability (Bak et al., 1987; Bak, 1996). Thus, the complex interactions between all these different homeostatic processes are important to bring the whole system into a stable state showing dynamics matching experimental data.

All of these developmental models consider the dynamics of axons and dendrites. However, as described above, also the dynamics of dendritic spines and axonal boutons are determined by homeostatic structural plasticity. Theoretical network models from the 1980s (Dammasch et al., 1986, 1988; Cromme and Dammasch, 1989) already showed that also such detailed models of homeostatic structural plasticity self-organize to reach a desired activity regime. Again, the resulting system is quite stable such that even the insertion of new neurons (by, for instance, neurogenesis in the hippocampus) does not perturb the global network state (Butz et al., 2008). Furthermore, by introducing a distance-dependency for forming new synapses, the network develops into a small-world network (Butz et al., 2014b).

The dynamics of these models can also be compared to *in vivo* measurements after input lesions or stroke-induced lesions (Butz et al., 2009; Butz and van Ooyen, 2013; Butz et al., 2014a). Interestingly, this comparison between *in vivo* and model dynamics enables conclusions on the activity-dependency of the different homeostatic processes. For instance, after a retinal lesion, neurons in the lesion projection zone (which have lost their external input) start to connect with active neurons at the border of the zone (Keck et al., 2008). In network models, this dynamics can only be seen if for small neuronal activations basically new dendritic spines are formed and axonal boutons are pruned (Butz and van Ooyen, 2013; Butz et al., 2014a). In contrast, if for small activities boutons are formed and spines are deleted, the system still reaches homeostasis, but the neurons in the lesion projection zone predominantly connect with each other and, thus, the whole zone decouples from the rest of the network (Butz and van Ooyen, 2013; Butz et al., 2014a).

Further predictions from these models are, for instance, that similar effects arise in networks with lesions after stroke (Butz et al., 2009, 2014a). Neurons affected by the deafferentiation (nearby the lesion zone) have problems in regaining their activity-homeostasis when the rest of the network is in homeostasis. This problem can be solved if, after stroke, the neurons, which are still in homeostasis, receive an external stimulation to trigger homeostatic structural plasticity and, thus, encourage rewiring. After this stimulation the whole network has reached homeostasis (Butz et al., 2009). Thus, studying the effects of structural plasticity also helps to gain insights in new potential medical treatments.

### Models of the interaction of hebbian and homeostatic structural plasticity

So far only a few models investigated the interaction between Hebbian and homeostatic structural plasticity (Levy and Desmond, 1985; Adelsberger-Mangan and Levy, 1993, 1994; Levy, 2004; Thomas et al., 2015). Basically, these models include weight-dependent synapse removal (Hebbian structural plasticity) with an activity-dependent synapse formation (homeostatic structural plasticity). The combination of these processes in a feed-forward network optimizes the information transfer from input to output layer and also supports the separation of information in the output layer while keeping the homeostasis (Adelsberger-Mangan and Levy, 1993, 1994). As already seen in developmental models, also in the combined models the assumption of different dynamics for axons and dendrites in homeostatic structural plasticity increases the overall performance of the network (Adelsberger-Mangan and Levy, 1994). In other words, this combination of Hebbian and homeostatic structural plasticity provides an unsupervised way to transfer, compress, and store information (Hebbian structural plasticity) in an energy efficient representation, i.e., with a low number of needed neurons and low firing rates (homeostatic structural plasticity). Along this line, each post-synaptic neuron becomes selective or tuned to a specific input pattern. The number of neurons tuned to one pattern grows with the occurrence of this pattern (Thomas et al., 2015). This could, in principle, be a solution to the problem of memory allocation or rather allocation of inputs to specific groups of neurons in the brain (Rogerson et al., 2014; Tetzlaff et al., 2015). These results provide first insights into the complex dynamics resulting from the interaction between Hebbian and homeostatic structural plasticity. However, further theoretical investigations are needed.

## Conclusions and open questions

**Figure 2:**
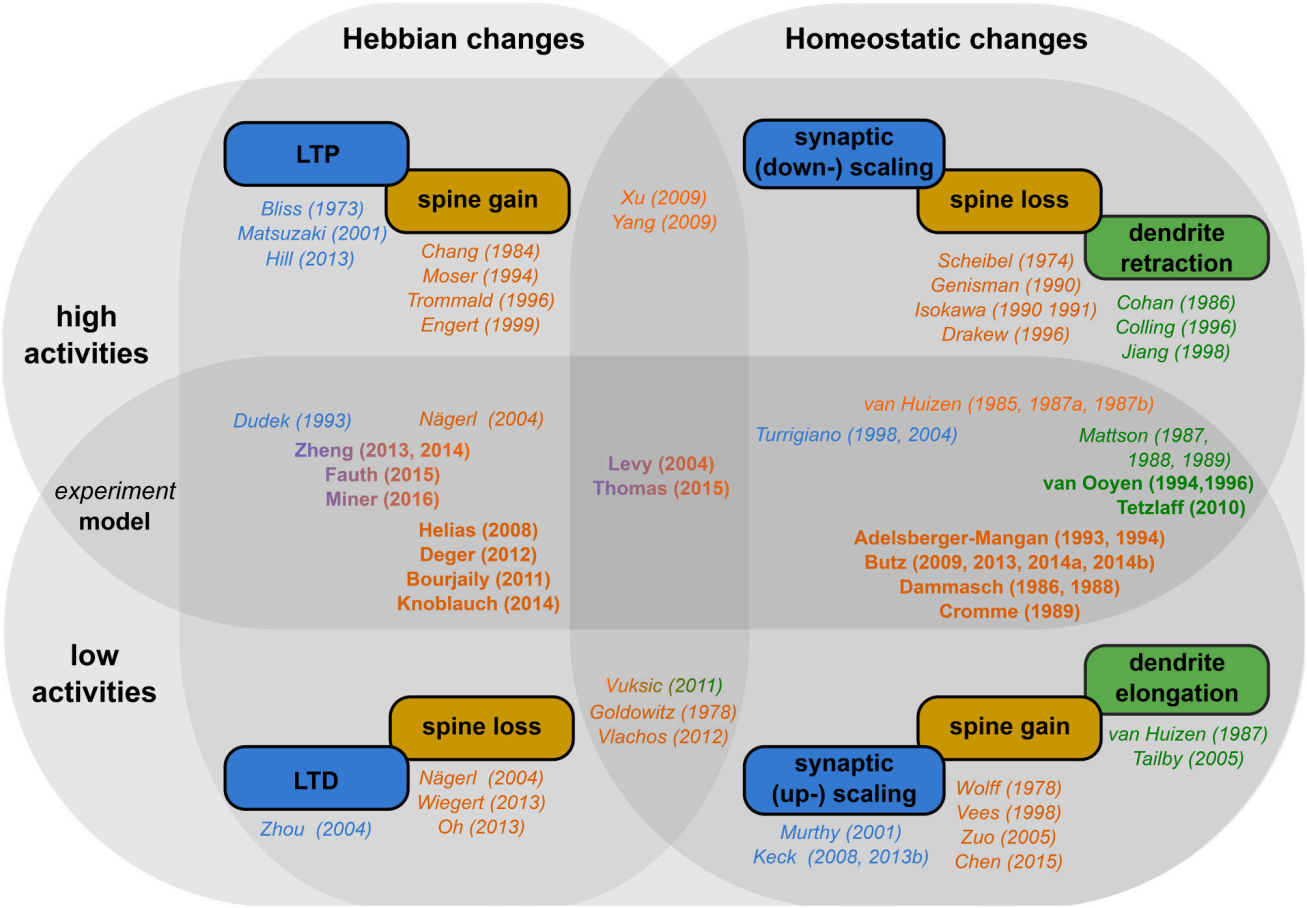
**Schematic overview of the literature**. *Rectangular boxes:* The different effects of activity-dependent plasticity for high activity (upper row) and low activity (lower row) in a Hebbian (first column) and a homeostatic manner (second column). *Colors* indicate, whether synaptic efficacies (blue), dendritic spines (orange), or axons and dendrites (green) are affected. *Names and years* outside the circles indicate key experimental (italic) and theoretical (bold) studies for the respective effect. Studies which target both activity regimes and/or plasticity types are placed in-between them.

In this review, we showed that structural plasticity can be classified into two categories (for a schematic summary, see Figure 2; italic and bold fonts indicate key references for experimental and theoretical studies respectively): (i) Hebbian structural plasticity leads to an increase (decrease) of the number or density of dendritic spines and contacts with axonal boutons during phases of high (low) activity (Figure 2, first column, orange). (ii) When these alterations in activity persist, homeostatic structural plasticity balances these changes by removing (adding new) synapses (Figure 2, second column, orange) and, after days, even by retracting (growing out) the dendrites themselves (Figure 2, second column, green).

In addition, we showed that there is a strong interaction between structural plasticity and synaptic plasticity. Both have been demonstrated to depend on (local) calcium levels. Even more strikingly, the synaptic transmission efficacies are related to the stability of the synapses. Thus, structural plasticity and its influences on the dynamics of neural networks can only be understood in conjunction with synaptic plasticity.

These complex interactions and their functional implications are best understood by theoretical models. For instance, Hebbian structural plasticity seems to remove and create synapses selectively. This selectivity leads to experimentally measured local connectivities and, furthermore, enhances memory lifetime, storage capacity, and robustness. For this, especially the pruning of non-needed synapses plays an important role. However, as for synaptic plasticity, these Hebbian dynamics lead to a positive feedback between connectivity and activity and, thus, to increasing neuronal activities. Thus, also structural plasticity requires a homeostatic process regulating activities back to an intermediate level. Accordingly, theoretical studies show that homeostatic structural plasticity organizes the connectivity of the network to maintain network stability. The combination of Hebbian and homeostatic structural plasticity preserves and improves network functions, as memory storage and input discrimination, and, in parallel, stabilizes the global dynamics in a resource efficient manner.

Still there are many open questions summarized in the following. On the experimental side, it is, for example, still unclear whether the increased number of spines after LTP-inducing stimuli results from increased stabilization or from an increased spine formation. Also, the relation between structural and synaptic plasticity is still not completely understood. Along these lines, especially the relation between homeostatic structural plasticity and synaptic scaling has not been completely unraveled yet.

Furthermore, we argued that the interaction of Hebbian and homeostatic structural plasticity will lead to a transient increase (decrease) of, e.g., the number of spines in a system which undergoes prolonged phases of enhanced (decreased) activity. Such transient increases are observed in experiments. However, experimental investigations, whether the dynamics occur due to this interaction, are still missing. In general, the interaction of Hebbian and homeostatic processes in the same system is difficult to tackle and has been addressed only by a few studies.

Accordingly, also most of the theoretical studies have focused on either Hebbian or homeostatic structural plasticity. The interaction of both mechanisms, especially in recurrent networks, is widely unknown. Moreover, theoretical studies are often restricted to either reproducing biological data or investigating functional consequences of structural plasticity. Therefore, more studies are needed to link experimentally obtained connectivity features to functional predictions.

The here reviewed theoretical models mostly considered point-neurons. However, the actual position or location of a synapse on the dendritic tree strongly influences the details of synaptic plasticity (Sjöström and Häusser, 2006). In addition, also neighborly relations between synapses influence via, for instance, calcium currents synaptic plasticity (Oh et al., 2015). Obviously, due to the interactions between synaptic and structural plasticity, these local influences on synaptic plasticity also affect structural plasticity. On the other side, structural plasticity might select synapses with certain synaptic plasticity rules and remove others. Thereby, structural plasticity could act as a metaplasticity-like process (Abraham, 2008) which adds another level of complexity to the interaction of the different plasticities.

Taken together, we already have a decent understanding of the basic mechanisms governing Hebbian and homeostatic structural changes. Yet, their interaction with each other and with synaptic plasticity, as well as their functional relevance, still leave many open questions.

## Author contributions

All authors listed, have made substantial, direct and intellectual contribution to the work, and approved it for publication.

## Funding

This research was supported by the Federal Ministry of Education and Research (BMBF) Germany under grant number 01GQ1005B [CT] and 01GQ1005A [MF], and by the Göttingen Graduate School for Neuroscience and Molecular Biosciences (DFG Grant GSC226/2) [MF].

### Conflict of interest statement

The authors declare that the research was conducted in the absence of any commercial or financial relationships that could be construed as a potential conflict of interest.

## References

[B1] Abraham W. C. (2008). Metaplasticity: tuning synapses and networks for plasticity. Nat. Rev. Neurosci. 9, 387–399. 10.1038/nrn2356 18401345

[B2] Adelsberger-Mangan D. M. Levy W. B. (1993). Adaptive synaptogenesis constructs networks that maintain information and reduce statistical dependence. Biol. Cybern. 70, 81–87. 10.1007/BF00202569 8312400

[B3] Adelsberger-Mangan D. M. Levy W. B. (1994). The influence of limited presynaptic growth and synapse removal on adaptive synaptogenesis. Biol. Cybern. 71, 461–468. 10.1007/BF00198922 7993933

[B4] Annis C. M. O'Dowd D. K. Robertson R. T. (1994). Activity-dependent regulation of dendritic spine density on cortical pyramidal neurons in organotypic slice cultures. J. Neurobiol. 25, 1483–1493. 7861113 10.1002/neu.480251202

[B5] Bak P. (1996). How Nature Works: The Science of Self-Organized Criticality, 1st Edn. New York, NY: Springer.

[B6] Bak P. Tang C. Wiesenfeld K. (1987). Self-organized criticality: an explanation of 1/f noise. Phys. Rev. Lett. 59, 381–384. 10.1103/PhysRevLett.59.381 10035754

[B7] Barker J. L. Behar T. Li Y. X. Liu Q. Y. Ma W. Maric D.. (1998). GABAergic cells and signals in CNS development. Perspect. Dev. Neurobiol. 5, 305–322. 9777645

[B8] Bastrikova N. Gardner G. A. Reece J. M. Jeromin A. Dudek S. M. (2008). Synapse elimination accompanies functional plasticity in hippocampal neurons. Proc. Natl. Acad. Sci. U.S.A. 105, 3123–3127. 10.1073/pnas.0800027105 18287055 PMC2268595

[B9] Beattie E. C. Carroll R. C. Yu X. Morishita W. Yasuda H. von Zastrow M.. (2000). Regulation of AMPA receptor endocytosis by a signaling mechanism shared with LTD. Nat. Neurosci. 3, 1291–1300. 10.1038/81823 11100150

[B10] Becker N. Wierenga C. J. Fonseca R. Bonhoeffer T. Nägerl U. V. (2008). LTD induction causes morphological changes of presynaptic boutons and reduces their contacts with spines. Neuron 60, 590–597. 10.1016/j.neuron.2008.09.018 19038217

[B11] Ben-Ari Y. (2002). Excitatory actions of GABA during development: the nature of the nurture. Nat. Rev. Neurosci. 3, 728–739. 10.1038/nrn920 12209121

[B12] Bi G. Q. Poo M. M. (1998). Synaptic modifications in cultured hippocampal neurons: dependence on spike timing, synaptic strength, and postsynaptic cell type. J. Neurosci. 18, 10464–10472. 9852584 10.1523/JNEUROSCI.18-24-10464.1998 PMC6793365

[B13] Bienenstock E. L. Cooper L. N. Munro P. W. (1982). Theory for the development of neuron selectivity: orientation specificity and binocular interaction in visual cortex. J. Neurosci. 2, 32–48. 7054394 10.1523/JNEUROSCI.02-01-00032.1982 PMC6564292

[B14] Bixby J. L. Grunwald G. B. Bookman R. J. (1994). Ca2+ influx and neurite growth in response to purified N-cadherin and laminin. J. Cell Biol. 127, 1461–1475. 10.1083/jcb.127.5.1461 7962102 PMC2120265

[B15] Bliss T. V. Lomo T. (1973). Long-lasting potentiation of synaptic transmission in the dentate area of the anaesthetized rabbit following stimulation of the perforant path. J. Physiol. 232, 331–356. 10.1113/jphysiol.1973.sp010273 4727084 PMC1350458

[B16] Bliss T. V. P. Collingridge G. L. (1993). A synaptic model of memory: long-term potentiation in the hippocampus. Nature 361, 31–39. 10.1038/361031a0 8421494

[B17] Bock J. Braun K. (1999). Blockade of N-methyl-D-aspartate receptor activation suppresses learning-induced synaptic elimination. Proc. Natl. Acad. Sci. U.S.A. 96, 2485–2490. 10.1073/pnas.96.5.2485 10051669 PMC26811

[B18] Bourjaily M. A. Miller P. (2011). Excitatory, inhibitory and structural plasticity produce correlated connectivity in random networks trained to solve paired-stimulus tasks. Front. Comput. Neurosci. 5:37. 10.3389/fncom.2011.00037 21991253 PMC3170885

[B19] Bourne J. N. Harris K. M. (2011). Coordination of size and number of excitatory and inhibitory synapses results in a balanced structural plasticity along mature hippocampal CA1 dendrites during LTP. Hippocampus 21, 354–373. 10.1002/hipo.20768 20101601 PMC2891364

[B20] Bourne J. N. Kirov S. A. Sorra K. E. Harris K. M. (2007). Warmer preparation of hippocampal slices prevents synapse proliferation that might obscure LTP-related structural plasticity. Neuropharmacology 52, 55–59. 10.1016/j.neuropharm.2006.06.020 16895730

[B21] Bravin M. Morando L. Vercelli A. Rossi F. Strata P. (1999). Control of spine formation by electrical activity in the adult rat cerebellum. Proc. Natl. Acad. Sci. U.S.A. 96, 1704–1709. 10.1073/pnas.96.4.1704 9990088 PMC15567

[B22] Burrone J. O'Byrne M. Murthy V. N. (2002). Multiple forms of synaptic plasticity triggered by selective suppression of activity in individual neurons. Nature 420, 414–418. 10.1038/nature01242 12459783

[B23] Butz M. Steenbuck I. D. van Ooyen A. (2014a). Homeostatic structural plasticity can account for topology changes following deafferentation and focal stroke. Front. Neuroanat. 8:115. 10.3389/fnana.2014.00115 25360087 PMC4199279

[B24] Butz M. Steenbuck I. D. van Ooyen A. (2014b). Homeostatic structural plasticity increases the efficiency of small-world networks. Front. Synaptic Neurosci. 6:7. 10.3389/fnsyn.2014.00007 24744727 PMC3978244

[B25] Butz M. Teuchert-Noodt G. Grafen K. van Ooyen A. (2008). Inverse relationship between adult hippocampal cell proliferation and synaptic rewiring in the dentate gyrus. Hippocampus 18, 879–898. 10.1002/hipo.20445 18481284

[B26] Butz M. van Ooyen A. (2013). A simple rule for dendritic spine and axonal bouton formation can account for cortical reorganization after focal retinal lesions. PLoS Comput. Biol. 9:e1003259. 10.1371/journal.pcbi.1003259 24130472 PMC3794906

[B27] Butz M. Wörgötter F. van Ooyen A. (2009). Activity-dependent structural plasticity. Brain Res. Rev. 60, 287–305. 10.1016/j.brainresrev.2008.12.023 19162072

[B28] Canty A. J. Huang L. Jackson J. S. Little G. E. Knott G. Maco B.. (2013). *In-vivo* single neuron axotomy triggers axon regeneration to restore synaptic density in specific cortical circuits. Nat. Commun. 4:2038. 10.1038/ncomms3038 23799397

[B29] Chang F. L. Greenough W. T. (1984). Transient and enduring morphological correlates of synaptic activity and efficacy change in the rat hippocampal slice. Brain Res. 309, 35–46. 10.1016/0006-8993(84)91008-4 6488013

[B30] Chen C.-C. Bajnath A. Brumberg J. C. (2015). The impact of development and sensory deprivation on dendritic protrusions in the mouse barrel cortex. Cereb. Cortex 25, 1638–1653. 10.1093/cercor/bht415 24408954 PMC4506326

[B31] Chiappalone M. Bove M. Vato A. Tedesco M. Martinoia S. (2006). Dissociated cortical networks show spontaneously correlated activity patterns during *in vitro* development. Brain Res. 1093, 41–53. 10.1016/j.brainres.2006.03.049 16712817

[B32] Chklovskii D. B. Mel B. W. Svoboda K. (2004). Cortical rewiring and information storage. Nature 431, 782–788. 10.1038/nature03012 15483599

[B33] Cohan C. S. Kater S. B. (1986). Suppression of neurite elongation and growth cone motility by electrical activity. Science 232, 1638–1640. 10.1126/science.3715470 3715470

[B34] Collin C. Miyaguchi K. Segal M. (1997). Dendritic spine density and LTP induction in cultured hippocampal slices. J. Neurophysiol. 77, 1614–1623. 9084624 10.1152/jn.1997.77.3.1614

[B35] Colling S. B. Man W. D. Draguhn A. Jefferys J. G. (1996). Dendritic shrinkage and dye-coupling between rat hippocampal CA1 pyramidal cells in the tetanus toxin model of epilepsy. Brain Res. 741, 38–43. 10.1016/S0006-8993(96)00884-0 9001702

[B36] Collingridge G. L. Peineau S. Howland J. G. Wang Y. T. (2010). Long-term depression in the CNS. Nat. Rev. Neurosci. 11, 459–473. 10.1038/nrn2867 20559335

[B37] Cromme L. J. Dammasch I. E. (1989). Compensation type algorithms for neural nets: stability and convergence. J. Math. Biol. 27, 327–340. 10.1007/BF00275816 2746141

[B38] Dalva M. B. Ghosh A. Shatz C. J. (1994). Independent control of dendritic and axonal form in the developing lateral geniculate nucleus. J. Neurosci. 14, 3588–3602. 8207474 10.1523/JNEUROSCI.14-06-03588.1994 PMC6576932

[B39] Dammasch I. E. Wagner G. P. Wolff J. R. (1986). Self-stabilization of neuronal networks. I. The compensation algorithm for synaptogenesis. Biol. Cybern. 54, 211–222. 10.1007/BF00318417 3017460

[B40] Dammasch I. E. Wagner G. P. Wolff J. R. (1988). Self-stabilization of neuronal networks. II. Stability conditions for synaptogenesis. Biol. Cybern. 58, 149–158. 10.1007/BF00364134 3358950

[B41] Darian-Smith C. Gilbert C. D. (1994). Axonal sprouting accompanies functional reorganization in adult cat striate cortex. Nature 368, 737–740. 10.1038/368737a0 8152484

[B42] Deger M. Helias M. Rotter S. Diesmann M. (2012). Spike-timing dependence of structural plasticity explains cooperative synapse formation in the neocortex. PLoS Comput. Biol. 8:e1002689. 10.1371/journal.pcbi.1002689 23028287 PMC3447982

[B43] Deller T. Bas Orth C. Vlachos A. Merten T. Del Turco D. Dehn D.. (2006). Plasticity of synaptopodin and the spine apparatus organelle in the rat fascia dentata following entorhinal cortex lesion. J. Comp. Neurol. 499, 471–484. 10.1002/cne.21103 16998909

[B44] Desmond N. L. Levy W. B. (1990). Morphological correlates of long-term potentiation imply the modification of existing synapses, not synaptogenesis, in the hippocampal dentate gyrus. Synapse 5, 139–143. 10.1002/syn.890050208 2309158

[B45] Drakew A. Müller M. Gähwiler B. H. Thompson S. M. Frotscher M. (1996). Spine loss in experimental epilepsy: quantitative light and electron microscopic analysis of intracellularly stained CA3 pyramidal cells in hippocampal slice cultures. Neuroscience 70, 31–45. 10.1016/0306-4522(95)00379-W 8848134

[B46] Dudai Y. (2004). The neurobiology of consolidations, or, how stable is the engram? Annu. Rev. Psychol. 55, 51–86. 10.1146/annurev.psych.55.090902.142050 14744210

[B47] Dudek S. M. Bear M. F. (1992). Homosynaptic long-term depression in area CA1 of hippocampus and effects of N-methyl-D-aspartate receptor blockade. Proc. Natl. Acad. Sci. U.S.A. 89, 4363–4367. 10.1073/pnas.89.10.4363 1350090 PMC49082

[B48] Dunaevsky A. Tashiro A. Majewska A. Mason C. Yuste R. (1999). Developmental regulation of spine motility in the mammalian central nervous system. Proc. Natl. Acad. Sci. U.S.A. 96, 13438–13443. 10.1073/pnas.96.23.13438 10557339 PMC23966

[B49] Engert F. Bonhoeffer T. (1999). Dendritic spine changes associated with hippocampal long-term synaptic plasticity. Nature 399, 66–70. 10.1038/19978 10331391

[B50] Fares T. Stepanyants A. (2009). Cooperative synapse formation in the neocortex. Proc. Natl. Acad. Sci. U.S.A. 106, 16463–16468. 10.1073/pnas.0813265106 19805321 PMC2738618

[B51] Fauth M. Wörgötter F. Tetzlaff C. (2015a). Formation and maintenance of robust long-term information storage in the presence of synaptic turnover. PLoS Comput. Biol. 11:e1004684. 10.1371/journal.pcbi.1004684 26713858 PMC4699846

[B52] Fauth M. Wörgötter F. Tetzlaff C. (2015b). The formation of multi-synaptic connections by the interaction of synaptic and structural plasticity and their functional consequences. PLoS Comput. Biol. 11:e1004031. 10.1371/journal.pcbi.1004031 25590330 PMC4295841

[B53] Feldman D. E. (2009). Synaptic mechanisms for plasticity in neocortex. Annu. Rev. Neurosci. 32, 33–55. 10.1146/annurev.neuro.051508.135516 19400721 PMC3071739

[B54] Feldman M. L. Dowd C. (1975). Loss of dendritic spines in aging cerebral cortex. Anat. Embryol. (Berl.) 148, 279–301. 10.1007/BF00319848 1221885

[B55] Feldmeyer D. Egger V. Lübke J. Sakmann B. (1999). Reliable synaptic connections between pairs of excitatory layer 4 neurones within a single 'barrel' of developing rat somatosensory cortex. J. Physiol. 521(Pt 1):169–190. 10.1111/j.1469-7793.1999.00169.x 10562343 PMC2269646

[B56] Feldmeyer D. Lübke J. Sakmann B. (2006). Efficacy and connectivity of intracolumnar pairs of layer 2/3 pyramidal cells in the barrel cortex of juvenile rats. J. Physiol. 575(Pt 2):583–602. 10.1113/jphysiol.2006.105106 16793907 PMC1819447

[B57] Feldmeyer D. Lübke J. Silver R. A. Sakmann B. (2002). Synaptic connections between layer 4 spiny neurone-layer 2/3 pyramidal cell pairs in juvenile rat barrel cortex: physiology and anatomy of interlaminar signalling within a cortical column. J. Physiol. 538(Pt 3):803–822. 10.1113/jphysiol.2001.012959 11826166 PMC2290091

[B58] Fields R. D. Neale E. A. Nelson P. G. (1990). Effects of patterned electrical activity on neurite outgrowth from mouse sensory neurons. J. Neurosci. 10, 2950–2964. 2398369 10.1523/JNEUROSCI.10-09-02950.1990 PMC6570242

[B59] Fifková E. Van Harreveld A. (1977). Long-lasting morphological changes in dendritic spines of dentate granular cells following stimulation of the entorhinal area. J. Neurocytol. 6, 211–230. 10.1007/BF01261506 856951

[B60] Flores C. E. Méndez P. (2014). Shaping inhibition: activity dependent structural plasticity of GABAergic synapses. Front. Cell. Neurosci. 8:327. 10.3389/fncel.2014.00327 25386117 PMC4209871

[B61] Friedman H. V. Bresler T. Garner C. C. Ziv N. E. (2000). Assembly of new individual excitatory synapses: time course and temporal order of synaptic molecule recruitment. Neuron 27, 57–69. 10.1016/S0896-6273(00)00009-X 10939331

[B62] Garyantes T. K. Regehr W. G. (1992). Electrical activity increases growth cone calcium but fails to inhibit neurite outgrowth from rat sympathetic neurons. J. Neurosci. 12, 96–103. 1729445 10.1523/JNEUROSCI.12-01-00096.1992 PMC6575705

[B63] Geinisman Y. Morrell F. deToledo Morrell L. (1990). Increase in the relative proportion of perforated axospinous synapses following hippocampal kindling is specific for the synaptic field of stimulated axons. Brain Res. 507, 325–331. 10.1016/0006-8993(90)90291-I 2337773

[B64] Gerstner W. Kempter R. van Hemmen J. L. Wagner H. (1996). A neuronal learning rule for sub-millisecond temporal coding. Nature 383, 76–78. 10.1038/383076a0 8779718

[B65] Gerstner W. Kistler W. M. (2002). Mathematical formulations of Hebbian learning. Biol. Cybern. 87, 404–415. 10.1007/s00422-002-0353-y 12461630

[B66] Goldowitz D. Scheff S. W. Cotman C. W. (1979). The specificity of reactive synaptogenesis: a comparative study in the adult rat hippocampal formation. Brain Res. 170, 427–441. 10.1016/0006-8993(79)90962-4 466422

[B67] Groc L. Petanjek Z. Gustafsson B. Ben-Ari Y. Hanse E. Khazipov R. (2002). *In vivo* blockade of neural activity alters dendritic development of neonatal CA1 pyramidal cells. Eur. J. Neurosci. 16, 1931–1938. 10.1046/j.1460-9568.2002.02264.x 12453057

[B68] Grutzendler J. Kasthuri N. Gan W.-B. (2002). Long-term dendritic spine stability in the adult cortex. Nature 420, 812–816. 10.1038/nature01276 12490949

[B69] Haas K. Li J. Cline H. T. (2006). AMPA receptors regulate experience-dependent dendritic arbor growth *in vivo*. Proc. Natl. Acad. Sci. U.S.A. 103, 12127–12131. 10.1073/pnas.0602670103 16882725 PMC1525049

[B70] Halpain S. Hipolito A. Saffer L. (1998). Regulation of F-actin stability in dendritic spines by glutamate receptors and calcineurin. J. Neurosci. 18, 9835–9844. 9822742 10.1523/JNEUROSCI.18-23-09835.1998 PMC6793298

[B71] Hardingham N. R. Read J. C. A. Trevelyan A. J. Nelson J. C. Jack J. J. B. Bannister N. J. (2010). Quantal analysis reveals a functional correlation between presynaptic and postsynaptic efficacy in excitatory connections from rat neocortex. J. Neurosci. 30, 1441–1451. 10.1523/JNEUROSCI.3244-09.2010 20107071 PMC2825095

[B72] Haydon P. G. McCobb D. P. Kater S. B. (1984). Serotonin selectively inhibits growth cone motility and synaptogenesis of specific identified neurons. Science 226, 561–564. 10.1126/science.6093252 6093252

[B73] Haydon P. G. McCobb D. P. Kater S. B. (1987). The regulation of neurite outgrowth, growth cone motility, and electrical synaptogenesis by serotonin. J. Neurobiol. 18, 197–215. 10.1002/neu.480180206 2883253

[B74] Hebb D. (1949). The Organization of Behavior: A Neuropsychological Theory. Wiley Book in Clinical Psychology. New York, NY: Wiley.

[B75] Helias M. Rotter S. Gewaltig M.-O. Diesmann M. (2008). Structural plasticity controlled by calcium based correlation detection. Front. Comput. Neurosci. 2:7. 10.3389/neuro.10.007.2008 19129936 PMC2614616

[B76] Helmchen F. Imoto K. Sakmann B. (1996). Ca2+ buffering and action potential-evoked Ca2+ signaling in dendrites of pyramidal neurons. Biophys. J. 70, 1069–1081. 10.1016/S0006-3495(96)79653-4 8789126 PMC1225009

[B77] Hengen K. B. Lambo M. E. Van Hooser S. D. Katz D. B. Turrigiano G. G. (2013). Firing rate homeostasis in visual cortex of freely behaving rodents. Neuron 80, 335–342. 10.1016/j.neuron.2013.08.038 24139038 PMC3816084

[B78] Higley M. J. Sabatini B. L. (2008). Calcium signaling in dendrites and spines: practical and functional considerations. Neuron 59, 902–913. 10.1016/j.neuron.2008.08.020 18817730

[B79] Hill T. C. Zito K. (2013). LTP-induced long-term stabilization of individual nascent dendritic spines. J. Neurosci. 33, 678–686. 10.1523/JNEUROSCI.1404-12.2013 23303946 PMC6704923

[B80] Hofer S. B. Mrsic-Flogel T. D. Bonhoeffer T. Hübener M. (2009). Experience leaves a lasting structural trace in cortical circuits. Nature 457, 313–317. 10.1038/nature07487 19005470 PMC6485433

[B81] Holtmaat A. J. G. D. Trachtenberg J. T. Wilbrecht L. Shepherd G. M. Zhang X. Knott G. W.. (2005). Transient and persistent dendritic spines in the neocortex *in vivo*. Neuron 45, 279–291. 10.1016/j.neuron.2005.01.003 15664179

[B82] Hopfield J. J. (1982). Neural networks and physical systems with emergent collective computational abilities. Proc. Natl. Acad. Sci. U.S.A. 79, 2554–2558. 10.1073/pnas.79.8.2554 6953413 PMC346238

[B83] Hou Q. Gilbert J. Man H. Y. (2011). Homeostatic regulation of AMPA receptor trafficking and degradation by light-controlled single-synaptic activation. Neuron 72, 806–818. 10.1016/j.neuron.2011.10.011 22153376 PMC3240854

[B84] Hou Q. Zhang D. Jarzylo L. Huganir R. L. Man H. Y. (2008). Homeostatic regulation of AMPA receptor expression at single hippocampal synapses. Proc. Natl. Acad. Sci. U.S.A. 105, 775–780. 10.1073/pnas.0706447105 18174334 PMC2206612

[B85] Hua J. Y. Smith S. J. (2004). Neural activity and the dynamics of central nervous system development. Nat. Neurosci. 7, 327–332. 10.1038/nn1218 15048120

[B86] Hübener M. Bonhoeffer T. (2010). Searching for engrams. Neuron 67, 363–371. 10.1016/j.neuron.2010.06.033 20696375

[B87] Hutchins B. I. Kalil K. (2008). Differential outgrowth of axons and their branches is regulated by localized calcium transients. J. Neurosci. 28, 143–153. 10.1523/JNEUROSCI.4548-07.2008 18171932 PMC2474798

[B88] Huttenlocher P. R. (1984). Synapse elimination and plasticity in developing human cerebral cortex. Am. J. Ment. Defic. 88, 488–496. 6731486

[B89] Huttenlocher P. R. de Courten C. Garey L. J. Van der Loos H. (1982). Synaptogenesis in human visual cortex–evidence for synapse elimination during normal development. Neurosci. Lett. 33, 247–252. 10.1016/0304-3940(82)90379-2 7162689

[B90] Ibata K. Sun Q. Turrigiano G. G. (2008). Rapid synaptic scaling induced by changes in postsynaptic firing. Neuron 57, 819–826. 10.1016/j.neuron.2008.02.031 18367083

[B91] Isokawa M. (1998). Remodeling dendritic spines in the rat pilocarpine model of temporal lobe epilepsy. Neurosci. Lett. 258, 73–76. 10.1016/S0304-3940(98)00848-9 9875530

[B92] Isokawa M. Levesque M. F. (1991). Increased NMDA responses and dendritic degeneration in human epileptic hippocampal neurons in slices. Neurosci. Lett. 132, 212–216. 10.1016/0304-3940(91)90304-C 1664504

[B93] Jiang M. Lee C. L. Smith K. L. Swann J. W. (1998). Spine loss and other persistent alterations of hippocampal pyramidal cell dendrites in a model of early-onset epilepsy. J. Neurosci. 18, 8356–8368. 9763479 10.1523/JNEUROSCI.18-20-08356.1998 PMC6792859

[B94] Kalisman N. Silberberg G. Markram H. (2005). The neocortical microcircuit as a tabula rasa. Proc. Natl. Acad. Sci. U.S.A. 102, 880–885. 10.1073/pnas.0407088102 15630093 PMC545526

[B95] Kappel D. Habenschuss S. Legenstein R. Maass W. (2015). Network plasticity as Bayesian inference. PLoS Comput. Biol. 11:e1004485. 10.1371/journal.pcbi.1004485 26545099 PMC4636322

[B96] Kasai H. Matsuzaki M. Noguchi J. Yasumatsu N. Nakahara H. (2003). Structure-stability-function relationships of dendritic spines. Trends Neurosci. 26, 360–368. 10.1016/S0166-2236(03)00162-0 12850432

[B97] Kater S. B. Mattson M. P. Guthrie P. B. (1989). Calcium-induced neuronal degeneration: a normal growth cone regulating signal gone awry (?). Ann. N. Y. Acad. Sci. 568, 252–261. 10.1111/j.1749-6632.1989.tb12514.x 2576508

[B98] Kauer J. A. Malenka R. C. Nicoll R. A. (1988). A persistent postsynaptic modification mediates long-term potentiation in the hippocampus. Neuron 1, 911–917. 10.1016/0896-6273(88)90148-1 2908443

[B99] Keck T. Keller G. B. Jacobsen R. I. Eysel U. T. Bonhoeffer T. Hübener M. (2013). Synaptic scaling and homeostatic plasticity in the mouse visual cortex *in vivo*. Neuron 80, 327–334. 10.1016/j.neuron.2013.08.018 24139037

[B100] Keck T. Mrsic-Flogel T. D. Vaz Alfonso M. Eysel U. T. Bonhoeffer T. Hübener M. (2008). Massive restructuring of neuronal circuits during functional reorganization of adult visual cortex. Nat. Neurosci. 11, 1162–1167. 10.1038/nn.2181 18758460

[B101] Kim J. Tsien R. W. Alger B. E. (2012). An improved test for detecting multiplicative homeostatic synaptic scaling. PLoS ONE 7:e37364. 10.1371/journal.pone.0037364 22615990 PMC3355135

[B102] Kirkwood A. Rioult M. G. Bear M. F. (1996). Experience-dependent modification of synaptic plasticity in visual cortex. Nature 381, 526–528. 10.1038/381526a0 8632826

[B103] Kirov S. A. Goddard C. A. Harris K. M. (2004). Age-dependence in the homeostatic upregulation of hippocampal dendritic spine number during blocked synaptic transmission. Neuropharmacology 47, 640–648. 10.1016/j.neuropharm.2004.07.039 15458835

[B104] Kirov S. A. Harris K. M. (1999). Dendrites are more spiny on mature hippocampal neurons when synapses are inactivated. Nat. Neurosci. 2, 878–883. 10.1038/13178 10491607

[B105] Kirov S. A. Sorra K. E. Harris K. M. (1999). Slices have more synapses than perfusion-fixed hippocampus from both young and mature rats. J. Neurosci. 19, 2876–2886. 10191305 10.1523/JNEUROSCI.19-08-02876.1999 PMC6782277

[B106] Knoblauch A. (2009). The role of structural plasticity and synaptic consolidation for memory and amnesia in a model of cortico-hippocampal interplay, in Connectionist Models of Behavior and Cognition II: Proceedings of the 11th Neural Computation and Psychology Workshop (Singapore: Wold Scientific Publishing), 79–90. 10.1142/9789812834232_0007

[B107] Knoblauch A. Körner E. Körner U. Sommer F. T. (2014). Structural synaptic plasticity has high memory capacity and can explain graded amnesia, catastrophic forgetting, and the spacing effect. PLoS ONE 9:e96485. 10.1371/journal.pone.0096485 24858841 PMC4032253

[B108] Knoblauch A. Palm G. Sommer F. T. (2009). Memory capacities for synaptic and structural plasticity. Neural Comput. 22, 289–341. 10.1162/neco.2009.08-07-588 19925281

[B109] Knott G. W. Holtmaat A. Wilbrecht L. Welker E. Svoboda K. (2006). Spine growth precedes synapse formation in the adult neocortex *in vivo*. Nat. Neurosci. 9, 1117–1124. 10.1038/nn1747 16892056

[B110] Kolodziejski C. Tetzlaff C. Wörgötter F. (2010). Closed-form treatment of the interactions between neuronal activity and timing-dependent plasticity in networks of linear neurons. Front. Comput. Neurosci. 4:134. 10.3389/fncom.2010.00134 21152348 PMC2998049

[B111] Kwon H.-B. Sabatini B. L. (2011). Glutamate induces de novo growth of functional spines in developing cortex. Nature 474, 100–104. 10.1038/nature09986 21552280 PMC3107907

[B112] Lamprecht R. LeDoux J. (2004). Structural plasticity and memory. Nat. Rev. Neurosci. 5, 45–54. 10.1038/nrn1301 14708003

[B113] Le Bé J.-V. Markram H. (2006). Spontaneous and evoked synaptic rewiring in the neonatal neocortex. Proc. Natl. Acad. Sci. U.S.A. 103, 13214–13219. 10.1073/pnas.0604691103 16924105 PMC1559779

[B114] Lee K. S. Schottler F. Oliver M. Lynch G. (1980). Brief bursts of high-frequency stimulation produce two types of structural change in rat hippocampus. J. Neurophysiol. 44, 247–258. 7411185 10.1152/jn.1980.44.2.247

[B115] Lee W.-C. A. Huang H. Feng G. Sanes J. R. Brown E. N. So P. T.. (2006). Dynamic remodeling of dendritic arbors in GABAergic interneurons of adult visual cortex. PLoS Biol. 4:e29. 10.1371/journal.pbio.0040029 16366735 PMC1318477

[B116] Leuner B. Falduto J. Shors T. J. (2003). Associative memory formation increases the observation of dendritic spines in the hippocampus. J. Neurosci. 23, 659–665. Available online at: http://www.jneurosci.org/content/23/2/659.long 12533625 10.1523/JNEUROSCI.23-02-00659.2003 PMC2740640

[B117] Levy W. B. (2004). Contrasting rules for synaptogenesis, modification of existing synapses, and synaptic removal as a function of neuronal computation. Neurocomputing 58, 343–350. 10.1016/j.neucom.2004.01.065

[B118] Levy W. B. Desmond N. L. (1985). The rules of elemental synaptic plasticity, in Synaptic Modification, Neuron Selectivity and Nervous System Organisation, eds Levy W. B. Anderson J. A. Lehmkuhle S. (Hillsdale, NJ: Lawrence Erlbaum Associates), 105–121.

[B119] Levy W. B. Steward O. (1983). Temporal contiguity requirements for long-term associative potentiation/depression in the hippocampus. Neuroscience 8, 791–797. 10.1016/0306-4522(83)90010-6 6306504

[B120] Lipton S. A. Kater S. B. (1989). Neurotransmitter regulation of neuronal outgrowth, plasticity and survival. Trends Neurosci. 12, 265–270. 10.1016/0166-2236(89)90026-X 2475939

[B121] Loewenstein Y. Yanover U. Rumpel S. (2015). Predicting the dynamics of network connectivity in the neocortex. J. Neurosci. 35, 12535–12544. 10.1523/JNEUROSCI.2917-14.2015 26354919 PMC6605403

[B122] Lohmann C. Finski A. Bonhoeffer T. (2005). Local calcium transients regulate the spontaneous motility of dendritic filopodia. Nat. Neurosci. 8, 305–312. 10.1038/nn1406 15711541

[B123] Lohmann C. Myhr K. L. Wong R. O. L. (2002). Transmitter-evoked local calcium release stabilizes developing dendrites. Nature 418, 177–181. 10.1038/nature00850 12110889

[B124] Lynch G. S. Dunwiddie T. Gribkoff V. (1977). Heterosynaptic depression: a postsynaptic correlate of long-term potentiation. Nature 266, 737–739. 10.1038/266737a0 195211

[B125] Lynch G. S. Larson J. Kelso S. Barrionuevo G. Schottler F. (1983). Intracellular injections of EGTA block induction of hippocampal long-term potentiation. Nature 305, 719–721. 10.1038/305719a0 6415483

[B126] Majewska A. K. Newton J. R. Sur M. (2006). Remodeling of synaptic structure in sensory cortical areas *in vivo*. J. Neurosci. 26, 3021–3029. 10.1523/JNEUROSCI.4454-05.2006 16540580 PMC6673961

[B127] Malenka R. C. Bear M. F. (2004). LTP and LTD: an embarrassment of riches. Neuron 44, 5–21. 10.1016/j.neuron.2004.09.012 15450156

[B128] Malenka R. C. Lancaster B. Zucker R. S. (1992). Temporal limits on the rise in postsynaptic calcium required for the induction of long-term potentiation. Neuron 9, 121–128. 10.1016/0896-6273(92)90227-5 1632966

[B129] Maletic-Savatic M. Malinow R. Svoboda K. (1999). Rapid dendritic morphogenesis in CA1 hippocampal dendrites induced by synaptic activity. Science 283, 1923–1927. 10.1126/science.283.5409.1923 10082466

[B130] Marder E. Goaillard J. M. (2006). Variability, compensation and homeostasis in neuron and network function. Nat. Rev. Neurosci. 7, 563–574. 10.1038/nrn1949 16791145

[B131] Marik S. A. Yamahachi H. McManus J. N. J. Szabo G. Gilbert C. D. (2010). Axonal dynamics of excitatory and inhibitory neurons in somatosensory cortex. PLoS Biol. 8:e1000395. 10.1371/journal.pbio.1000395 20563307 PMC2885981

[B132] Markram H. Lübke J. Frotscher M. Roth A. Sakmann B. (1997a). Physiology and anatomy of synaptic connections between thick tufted pyramidal neurones in the developing rat neocortex. J. Physiol. 500(Pt 2), 409–440. 10.1113/jphysiol.1997.sp022031 9147328 PMC1159394

[B133] Markram H. Lübke J. Frotscher M. Sakmann B. (1997b). Regulation of synaptic efficacy by coincidence of postsynaptic APs and EPSPs. Science 275, 213–215. 10.1126/science.275.5297.213 8985014

[B134] Markram H. Gerstner W. Sjöström P. J. (2011). A history of spike-timing-dependent plasticity. Front. Synaptic Neurosci. 3:4. 10.3389/fnsyn.2011.00004 22007168 PMC3187646

[B135] Martin S. J. Grimwood P. D. Morris R. G. (2000). Synaptic plasticity and memory: an evaluation of the hypothesis. Annu. Rev. Neurosci. 23, 649–711. 10.1146/annurev.neuro.23.1.649 10845078

[B136] Matsuzaki M. Ellis-Davies G. C. Nemoto T. Miyashita Y. Iino M. Kasai H. (2001). Dendritic spine geometry is critical for AMPA receptor expression in hippocampal CA1 pyramidal neurons. Nat. Neurosci. 4:1086–1092. 10.1038/nn736 11687814 PMC4229049

[B137] Matsuzaki M. Honkura N. Ellis-Davies G. C. R. Kasai H. (2004). Structural basis of long-term potentiation in single dendritic spines. Nature 429, 761–766. 10.1038/nature02617 15190253 PMC4158816

[B138] Mattson M. P. (1988). Neurotransmitters in the regulation of neuronal cytoarchitecture. Brain Res. 472, 179–212. 10.1016/0165-0173(88)90020-3 2898278

[B139] Mattson M. P. Dou P. Kater S. B. (1988). Outgrowth-regulating actions of glutamate in isolated hippocampal pyramidal neurons. J. Neurosci. 8, 2087–2100. 2898515 10.1523/JNEUROSCI.08-06-02087.1988 PMC6569320

[B140] Mattson M. P. Kater S. B. (1987). Calcium regulation of neurite elongation and growth cone motility. J. Neurosci. 7, 4034–4043. 3121806 10.1523/JNEUROSCI.07-12-04034.1987 PMC6569087

[B141] Mattson M. P. Kater S. B. (1989). Excitatory and inhibitory neurotransmitters in the generation and degeneration of hippocampal neuroarchitecture. Brain Res. 478, 337–348. 10.1016/0006-8993(89)91514-X 2564301

[B142] McAllister A. K. Katz L. C. Lo D. C. (1996). Neurotrophin regulation of cortical dendritic growth requires activity. Neuron 17, 1057–1064. 10.1016/S0896-6273(00)80239-1 8982155

[B143] Medvedev N. I. Dallérac G. Popov V. I. Rodriguez Arellano J. J. Davies H. A. Kraev I. V.. (2014). Multiple spine boutons are formed after long-lasting LTP in the awake rat. Brain Struct. Funct. 219, 407–414. 10.1007/s00429-012-0488-0 23224218

[B144] Miller K. D. (1996). Synaptic economics: competition and cooperation in synaptic plasticity. Neuron 17, 371–374. 10.1016/S0896-6273(00)80169-5 8816700

[B145] Milo R. Shen-Orr S. Itzkovitz S. Kashtan N. Chklovskii D. Alon U. (2002). Network motifs: simple building blocks of complex networks. Science 298, 824–827. 10.1126/science.298.5594.824 12399590

[B146] Miner D. Triesch J. (2016). Plasticity-driven self-organization under topological constraints aaccount for nonrandom features of cortical synaptic wiring. PLoS Comput. Biol. 12:e1004759. 10.1371/journal.pcbi.1004759 26866369 PMC4750861

[B147] Miquelajauregui A. Kribakaran S. Mostany R. Badaloni A. Consalez G. G. Portera-Cailliau C. (2015). Layer 4 pyramidal neurons exhibit robust dendritic spine plasticity *in vivo* after input deprivation. J. Neurosci. 35, 7287–7294. 10.1523/JNEUROSCI.5215-14.2015 25948276 PMC4420789

[B148] Mizrahi A. Crowley J. C. Shtoyerman E. Katz L. C. (2004). High-resolution *in vivo* imaging of hippocampal dendrites and spines. J. Neurosci. 24, 3147–3151. 10.1523/JNEUROSCI.5218-03.2004 15056694 PMC6730023

[B149] Morris R. G. M. Anderson E. Lynch G. S. Baudry M. (1986). Selective impairment of learning and blockade of long-term potentiation by an N-methyl-D-aspartate receptor antagonist, AP5. Nature 319, 774–776. 10.1038/319774a0 2869411

[B150] Moser M. B. Trommald M. Andersen P. (1994). An increase in dendritic spine density on hippocampal CA1 pyramidal cells following spatial learning in adult rats suggests the formation of new synapses. Proc. Natl. Acad. Sci. U.S.A. 91, 12673–12675. 10.1073/pnas.91.26.12673 7809099 PMC45501

[B151] Mulkey R. M. Malenka R. C. (1992). Mechanisms underlying induction of homosynaptic long-term depression in area CA1 of the hippocampus. Neuron 9, 967–975. 10.1016/0896-6273(92)90248-C 1419003

[B152] Muller D. Lynch G. (1988). Long-term potentiation differentially affects two components of synaptic responses in hippocampus. Proc. Natl. Acad. Sci. U.S.A. 85, 9346–9350. 10.1073/pnas.85.23.9346 2904150 PMC282736

[B153] Müller M. Gähwiler B. H. Rietschin L. Thompson S. M. (1993). Reversible loss of dendritic spines and altered excitability after chronic epilepsy in hippocampal slice cultures. Proc. Natl. Acad. Sci. U.S.A. 90, 257–261. 10.1073/pnas.90.1.257 8093558 PMC45639

[B154] Murthy V. N. Schikorski T. Stevens C. F. Zhu Y. (2001). Inactivity produces increases in neurotransmitter release and synapse size. Neuron 32, 673–682. 10.1016/S0896-6273(01)00500-1 11719207

[B155] Nägerl U. V. Eberhorn N. Cambridge S. B. Bonhoeffer T. (2004). Bidirectional activity-dependent morphological plasticity in hippocampal neurons. Neuron 44, 759–767. 10.1016/j.neuron.2004.11.016 15572108

[B156] Nägerl U. V. Köstinger G. Anderson J. C. Martin K. A. C. Bonhoeffer T. (2007). Protracted synaptogenesis after activity-dependent spinogenesis in hippocampal neurons. J. Neurosci. 27, 8149–8156. 10.1523/JNEUROSCI.0511-07.2007 17652605 PMC6672732

[B157] Nägerl U. V. Willig K. I. Hein B. Hell S. W. Bonhoeffer T. (2008). Live-cell imaging of dendritic spines by STED microscopy. Proc. Natl. Acad. Sci. U.S.A. 105, 18982–18987. 10.1073/pnas.0810028105 19028874 PMC2585941

[B158] Nakayama K. Kiyosue K. Taguchi T. (2005). Diminished neuronal activity increases neuron-neuron connectivity underlying silent synapse formation and the rapid conversion of silent to functional synapses. J. Neurosci. 25, 4040–4051. 10.1523/JNEUROSCI.4115-04.2005 15843606 PMC6724957

[B159] Nikonenko I. Jourdain P. Muller D. (2003). Presynaptic remodeling contributes to activity-dependent synaptogenesis. J. Neurosci. 23, 8498–8505. Available online at: http://www.jneurosci.org/content/23/24/8498.long 13679418 10.1523/JNEUROSCI.23-24-08498.2003 PMC6740377

[B160] Ninan I. Liu S. Rabinowitz D. Arancio O. (2006). Early presynaptic changes during plasticity in cultured hippocampal neurons. EMBO J. 25, 4361–4371. 10.1038/sj.emboj.7601318 16957772 PMC1570425

[B161] Oh W. C. Hill T. C. Zito K. (2013). Synapse-specific and size-dependent mechanisms of spine structural plasticity accompanying synaptic weakening. Proc. Natl. Acad. Sci. U.S.A. 110, E305–E312. 10.1073/pnas.1214705110 23269840 PMC3557099

[B162] Oh W. C. Parajuli L. K. Zito K. (2015). Heterosynaptic structural plasticity on local dendritic segments of hippocampal CA1 neurons. Cell Rep. 10, 162–169. 10.1016/j.celrep.2014.12.016 25558061 PMC4294981

[B163] Oja E. (1982). A simplified neuron model as a principal component analyzer. J. Math. Biol. 15, 267–273. 10.1007/BF00275687 7153672

[B164] Okamoto K.-I. Nagai T. Miyawaki A. Hayashi Y. (2004). Rapid and persistent modulation of actin dynamics regulates postsynaptic reorganization underlying bidirectional plasticity. Nat. Neurosci. 7, 1104–1112. 10.1038/nn1311 15361876

[B165] Paola V. D. Holtmaat A. Knott G. Song S. Wilbrecht L. Caroni P.. (2006). Cell type-specific structural plasticity of axonal branches and boutons in the adult neocortex. Neuron 49, 861–875. 10.1016/j.neuron.2006.02.017 16543134

[B166] Parnavelas J. G. Lynch G. Brecha N. Cotman C. W. Globus A. (1974). Spine loss and regrowth in hippocampus following deafferentation. Nature 248, 71–73. 10.1038/248071a0 4818565

[B167] Pastalkova E. Serrano P. Pinkhasova D. Wallace E. Fenton A. A. Sacktor T. C. (2006). Storage of spatial information by the maintaince mechanism of LTP. Science 313, 1141–1144. 10.1126/science.1128657 16931766

[B168] Paul L. A. Scheibel A. B. (1986). Structural substrates of epilepsy. Adv. Neurol. 44, 775–786. 3706025

[B169] Perin R. Berger T. K. Markram H. (2011). A synaptic organizing principle for cortical neuronal groups. Proc. Natl. Acad. Sci. U.S.A. 108, 5419–5424. 10.1073/pnas.1016051108 21383177 PMC3069183

[B170] Platschek S. Cuntz H. Vuksic M. Deller T. Jedlicka P. (2016). A general homeostatic principle following lesion induced dendritic remodeling. Acta Neuropathol. Commun. 4:19. 10.1186/s40478-016-0285-8 26916562 PMC4766619

[B171] Portera-Cailliau C. Pan D. T. Yuste R. (2003). Activity-regulated dynamic behavior of early dendritic protrusions: evidence for different types of dendritic filopodia. J. Neurosci. 23, 7129–7142. Available online at: http://www.jneurosci.org/content/23/18/7129.long 12904473 10.1523/JNEUROSCI.23-18-07129.2003 PMC6740658

[B172] Ramakers G. J. Corner M. A. Habets A. M. (1990). Development in the absence of spontaneous bioelectric activity results in increased stereotyped burst firing in cultures of dissociated cerebral cortex. Exp. Brain Res. 79, 157–166. 10.1007/BF00228885 2311692

[B173] Riedel H. Schild D. (1992). The dynamics of Hebbian synapses can be stabilized by a nonlinear decay term. Neural Netw. 5, 459–463. 10.1016/0893-6080(92)90007-6

[B174] Rioult-Pedotti M.-S. Friedman D. Hess G. Donoghue J. P. (1998). Strengthening of horizontal cortical connections following skill learning. Nat. Neurosci. 1, 230–234. 10.1038/678 10195148

[B175] Rocha M. Sur M. (1995). Rapid acquisition of dendritic spines by visual thalamic neurons after blockade of N-methyl-D-aspartate receptors. Proc. Natl. Acad. Sci. U.S.A. 92, 8026–8030. 10.1073/pnas.92.17.8026 7644532 PMC41279

[B176] Rochester N. Holland J. Haibt L. Duda W. (1956). Tests on a cell assembly theory of the action of the brain, using a large digital computer. IRE Trans. Inf. Theory 2, 80–93. 10.1109/TIT.1956.1056810

[B177] Rogerson T. Cai D. J. Frank A. Sano Y. Shobe J. Lopez-Aranda M. F.. (2014). Synaptic tagging during memory allocation. Nat. Rev. Neurosci. 15, 157–169. 10.1038/nrn3667 24496410 PMC3992944

[B178] Ruthazer E. S. Li J. Cline H. T. (2006). Stabilization of axon branch dynamics by synaptic maturation. J. Neurosci. 26, 3594–3603. 10.1523/JNEUROSCI.0069-06.2006 16571768 PMC6673865

[B179] Scheibel M. E. Crandall P. H. Scheibel A. B. (1974). The hippocampal-dentate complex in temporal lobe epilepsy. A golgi study. Epilepsia 15, 55–80. 10.1111/j.1528-1157.1974.tb03997.x 4523024

[B180] Segal I. Korkotian I. Murphy D. D. (2000). Dendritic spine formation and pruning: common cellular mechanisms? Trends Neurosci. 23, 53–57. 10.1016/S0166-2236(99)01499-X 10652540

[B181] Segal M. (2005). Dendritic spines and long-term plasticity. Nat. Rev. Neurosci. 6, 277–284. 10.1038/nrn1649 15803159

[B182] Sejnowski T. J. (1977a). Statistical constraints on synaptic plasticity. J. Theor. Biol. 69, 385–389. 10.1016/0022-5193(77)90146-1 592884

[B183] Sejnowski T. J. (1977b). Storing covariance with nonlinearly interacting neurons. J. Math. Biol. 4, 303–321. 10.1007/BF00275079 925522

[B184] Shi S.-H. Hayashi Y. Petralia R. S. Zaman S. H. Wenthold R. J. Svoboda K.. (1999). Rapid spine delivery and redistribution of AMPA receptors after synaptic NMDA receptor activation. Science 284, 1811–1816. 10.1126/science.284.5421.1811 10364548

[B185] Sin W. C. Haas K. Ruthazer E. S. Cline H. T. (2002). Dendrite growth increased by visual activity requires NMDA receptor and Rho GTPases. Nature 419, 475–480. 10.1038/nature00987 12368855

[B186] Sjöström P. J. Häusser M. (2006). A cooperative switch determines the sign of synaptic plasticity in distal dendrites of neocortical pyramidal neurons. Neuron 51, 227–238. 10.1016/j.neuron.2006.06.017 16846857 PMC7616902

[B187] Song S. Sjöström P. J. Reigl M. Nelson S. Chklovskii D. B. (2005). Highly nonrandom features of synaptic connectivity in local cortical circuits. PLoS Biol. 3:e68. 10.1371/journal.pbio.0030068 15737062 PMC1054880

[B188] Sorra K. E. Harris K. M. (1998). Stability in synapse number and size at 2 hr after long-term potentiation in hippocampal area CA1. J. Neurosci. 18, 658–671. 9425008 10.1523/JNEUROSCI.18-02-00658.1998 PMC6792539

[B189] Spruston N. Schiller Y. Stuart G. Sakmann B. (1995). Activity-dependent action potential invasion and calcium influx into hippocampal CA1 dendrites. Science 268, 297–300. 10.1126/science.7716524 7716524

[B190] Stettler D. D. Yamahachi H. Li W. Denk W. Gilbert C. D. (2006). Axons and synaptic boutons are highly dynamic in adult visual cortex. Neuron 49, 877–887. 10.1016/j.neuron.2006.02.018 16543135

[B191] Stevens C. F. Wang Y. (1994). Changes in reliability of synaptic function as a mechanism for plasticity. Nature 371, 704–707. 10.1038/371704a0 7935816

[B192] Stoop R. Poo M. M. (1996). Synaptic modulation by neurotrophic factors: differential and synergistic effects of brain-derived neurotrophic factor and ciliary neurotrophic factor. J. Neurosci. 16, 3256–3264. 8627363 10.1523/JNEUROSCI.16-10-03256.1996 PMC6579145

[B193] Tailby C. Wright L. L. Metha A. B. Calford M. B. (2005). Activity-dependent maintenance and growth of dendrites in adult cortex. Proc. Natl. Acad. Sci. U.S.A. 102, 4631–4636. 10.1073/pnas.0402747102 15767584 PMC555467

[B194] Tetzlaff C. Dasgupta S. Kulvicius T. Wörgötter F. (2015). The use of Hebbian cell assemblies for nonlinear computation. Sci. Rep. 5:12866. 10.1038/srep12866 26249242 PMC4650703

[B195] Tetzlaff C. Kolodziejski C. Timme M. Wörgötter F. (2011). Synaptic scaling in combination with many generic plasticity mechanisms stabilizes circuit connectivity. Front. Comput. Neurosci. 5:47. 10.3389/fncom.2011.00047 22203799 PMC3214727

[B196] Tetzlaff C. Okujeni S. Egert U. Wörgötter F. Butz M. (2010). Self-organized criticality in developing neuronal networks. PLoS Comput. Biol. 6:e1001013. 10.1371/journal.pcbi.1001013 21152008 PMC2996321

[B197] Thomas B. T. Blalock D. W. Levy W. B. (2015). Adaptive synaptogenesis constructs neural codes that benefit discrimination. PLoS Comput. Biol. 11:e1004299. 10.1371/journal.pcbi.1004299 26176744 PMC4503424

[B198] Tian X. Kai L. Hockberger P. E. Wokosin D. L. Surmeier D. J. (2010). MEF-2 regulates activity-dependent spine loss in striatopallidal medium spiny neurons. Mol. Cell. Neurosci. 44, 94–108. 10.1016/j.mcn.2010.01.012 20197093 PMC2878643

[B199] Toni N. Buchs P. A. Nikonenko I. Bron C. R. Muller D. (1999). LTP promotes formation of multiple spine synapses between a single axon terminal and a dendrite. Nature 402, 421–425. 10.1038/46574 10586883

[B200] Tønnesen J. Katona G. Rózsa B. Nägerl U. V. (2014). Spine neck plasticity regulates compartmentalization of synapses. Nat. Neurosci. 17, 678–685. 10.1038/nn.3682 24657968

[B201] Tønnesen J. Nadrigny F. Willig K. I. Wedlich-Söldner R. Nägerl U. V. (2011). Two-color STED microscopy of living synapses using a single laser-beam pair. Biophys. J. 101, 2545–2552. 10.1016/j.bpj.2011.10.011 22098754 PMC3218326

[B202] Toyoizumi T. Kaneko M. Stryker M. P. Miller K. D. (2014). Modeling the dynamic interaction of Hebbian and homeostatic plasticity. Neuron 84, 497–510. 10.1016/j.neuron.2014.09.036 25374364 PMC4223656

[B203] Trachtenberg J. T. Chen B. E. Knott G. W. Feng G. Sanes J. R. Welker E.. (2002). Long-term *in vivo* imaging of experience-dependent synaptic plasticity in adult cortex. Nature 420, 788–794. 10.1038/nature01273 12490942

[B204] Trommald M. Hulleberg G. Andersen P. (1996). Long-term potentiation is associated with new excitatory spine synapses on rat dentate granule cells. Learn. Mem. 3, 218–228. 10.1101/lm.3.2-3.218 10456092

[B205] Turrigiano G. G. (2008). The self-tuning neuron: synaptic scaling of excitatory synapses. Cell 135, 422–435. 10.1016/j.cell.2008.10.008 18984155 PMC2834419

[B206] Turrigiano G. G. (2011). Too many cooks? Intrinsic and synaptic homeostatic mechanisms in cortical circuit refinement. Annu. Rev. Neurosci. 34, 89–103. 10.1146/annurev-neuro-060909-153238 21438687

[B207] Turrigiano G. G. Leslie K. R. Desai N. S. Rutherford L. C. Nelson S. B. (1998). Activity-dependent scaling of quantal amplitude in neocortical neurons. Nature 391, 892–896. 10.1038/36103 9495341

[B208] Turrigiano G. G. Nelson S. B. (2004). Homeostatic plasticity in the developing nervous system. Nat. Rev. Neurosci. 5, 97–107. 10.1038/nrn1327 14735113

[B209] Vaillant A. R. Zanassi P. Walsh G. S. Aumont A. Alonso A. Miller F. D. (2002). Signaling mechanisms underlying reversible, activity-dependent dendrite formation. Neuron 34, 985–998. 10.1016/S0896-6273(02)00717-1 12086645

[B210] van Huizen F. Romijn H. J. (1987). Tetrodotoxin enhances initial neurite outgrowth from fetal rat cerebral cortex cells *in vitro*. Brain Res. 408, 271–274. 10.1016/0006-8993(87)90386-6 3594216

[B211] van Huizen F. Romijn H. J. Habets A. M. (1985). Synaptogenesis in rat cerebral cortex cultures is affected during chronic blockade of spontaneous bioelectric activity by tetrodotoxin. Brain Res. 351, 67–80. 10.1016/0165-3806(85)90232-9 3995341

[B212] van Huizen F. Romijn H. J. Habets A. M. van den Hooff P. (1987). Accelerated neural network formation in rat cerebral cortex cultures chronically disinhibited with picrotoxin. Exp. Neurol. 97, 280–288. 10.1016/0014-4886(87)90089-6 3609212

[B213] van Ooyen A. (ed.). (2003). Modeling Neural Development. Cambridge: MIT Press.

[B214] van Ooyen A. (2011). Using theoretical models to analyse neural development. Nat. Rev. Neurosci. 12, 311–326. 10.1038/nrn3031 21587288

[B215] van Ooyen A. van Pelt J. (1994). Activity-dependent outgrowth of neurons and overshoot phenomena in developing neural networks. J. Theor. Biol. 167, 27–43. 10.1006/jtbi.1994.1047

[B216] van Ooyen A. van Pelt J. (1996). Complex periodic behaviour in a neural network model with activity-dependent neurite outgrowth. J. Theor. Biol. 179, 229–242. 10.1006/jtbi.1996.0063 8762335

[B217] van Ooyen A. van Pelt J. Corner M. A. (1995). Implications of activity dependent neurite outgrowth for neuronal morphology and network development. J. Theor. Biol. 172, 63–82. 10.1006/jtbi.1995.0005 7891450

[B218] van Pelt J. van Ooyen A. Corner M. A. (1996). Growth cone dynamics and activity-dependent processes in neuronal network development. Prog. Brain Res. 108, 333–346. 10.1016/S0079-6123(08)62550-9 8979812

[B219] Vees A. M. Micheva K. D. Beaulieu C. Descarries L. (1998). Increased number and size of dendritic spines in ipsilateral barrel field cortex following unilateral whisker trimming in postnatal rat. J. Comp. Neurol. 400, 110–124. 9762870

[B220] Vitureira N. Goda Y. (2013). The interplay between Hebbian and homeostatic synaptic plasticity. J. Cell Biol. 203, 175–186. 10.1083/jcb.201306030 24165934 PMC3812972

[B221] Vlachos A. Becker D. Jedlicka P. Winkels R. Roeper J. Deller T. (2012a). Entorhinal denervation induces homeostatic synaptic scaling of excitatory postsynapses of dentate granule cells in mouse organotypic slice cultures. PLoS ONE 7:e32883. 10.1371/journal.pone.0032883 22403720 PMC3293910

[B222] Vlachos A. Helias M. Becker D. Diesmann M. Deller T. (2013). NMDA-receptor inhibition increases spine stability of denervated mouse dentate granule cells and accelerates spine density recovery following entorhinal denervation *in vitro*. Neurobiol. Dis. 59, 267–276. 10.1016/j.nbd.2013.07.018 23932917

[B223] Vlachos A. Orth C. B. Schneider G. Deller T. (2012b). Time-lapse imaging of granule cells in mouse entorhino-hippocampal slice cultures reveals changes in spine stability after entorhinal denervation. J. Comp. Neurol. 520, 1891–1902. 10.1002/cne.23017 22134835

[B224] Vogels T. P. Frömke R. C. Doyon N. Gilson M. Haas J. S. Liu R.. (2013). Inhibitory synaptic plasticity: spike timing-dependence and putative network function. Front. Neural Circuits 7:119. 10.3389/fncir.2013.00119 23882186 PMC3714539

[B225] von der Malsburg C. (1973). Self-organization of orientation sensitive cells in the striate cortex. Kybernetik 14, 85–100. 10.1007/BF00288907 4786750

[B226] Vuksic M. Turco D. D. Vlachos A. Schuldt G. Müller C. M. Schneider G.. (2011). Unilateral entorhinal denervation leads to long-lasting dendritic alterations of mouse hippocampal granule cells. Exp. Neurol. 230, 176–185. 10.1016/j.expneurol.2011.04.011 21536031

[B227] Wagenaar D. A. Pine J. Potter S. M. (2006). An extremely rich repertoire of bursting patterns during the development of cortical cultures. BMC Neurosci. 7:11. 10.1186/1471-2202-7-11 16464257 PMC1420316

[B228] Whitlock J. R. Heynen A. J. Shuler M. G. Bear M. F. (2006). Learning induces long-term potentiation in the hippocampus. Science 313, 1093–1097. 10.1126/science.1128134 16931756

[B229] Wiegert J. S. Oertner T. G. (2013). Long-term depression triggers the selective elimination of weakly integrated synapses. Proc. Natl. Acad. Sci. U.S.A. 110, E4510–E4519. 10.1073/pnas.1315926110 24191047 PMC3839749

[B230] Willshaw D. J. Buneman O. P. Longuet-Higgins H. C. (1969). Non-holographic associative memory. Nature 222, 960–962. 10.1038/222960a0 5789326

[B231] Wolff J. R. Joó F. Dames W. (1978). Plasticity in dendrites shown by continuous GABA administration in superior cervical ganglion of adult rat. Nature 274, 72–74. 10.1038/274072a0 661998

[B232] Wong R. O. L. Ghosh A. (2002). Activity-dependent regulation of dendritic growth and patterning. Nat. Rev. Neurosci. 3, 803–812. 10.1038/nrn941 12360324

[B233] Wu G. Y. Cline H. T. (1998). Stabilization of dendritic arbor structure *in vivo* by CaMKII. Science 279, 222–226. 10.1126/science.279.5348.222 9422694

[B234] Xu T. Yu X. Perlik A. J. Tobin W. F. Zweig J. A. Tennant K.. (2009). Rapid formation and selective stabilization of synapses for enduring motor memories. Nature 462, 915–919. 10.1038/nature08389 19946267 PMC2844762

[B235] Yamahachi H. Marik S. A. McManus J. N. J. Denk W. Gilbert C. D. (2009). Rapid axonal sprouting and pruning accompany functional reorganization in primary visual cortex. Neuron 64, 719–729. 10.1016/j.neuron.2009.11.026 20005827 PMC2818836

[B236] Yang G. Pan F. Gan W.-B. (2009). Stably maintained dendritic spines are associated with lifelong memories. Nature 462, 920–924. 10.1038/nature08577 19946265 PMC4724802

[B237] Yang Y. Wang X. B. Frerking M. Zhou Q. (2008). Spine expansion and stabilization associated with long-term potentiation. J. Neurosci. 28, 5740–5751. 10.1523/JNEUROSCI.3998-07.2008 18509035 PMC2561912

[B238] Yasumatsu N. Matsuzaki M. Miyazaki T. Noguchi J. Kasai H. (2008). Principles of long-term dynamics of dendritic spines. J. Neurosci. 28, 13592–13608. 10.1523/JNEUROSCI.0603-08.2008 19074033 PMC2706274

[B239] Yin J. Yuan Q. (2014). Structural homeostasis in the nervous system: a balancing act for wiring plasticity and stability. Front. Cell. Neurosci. 8:439. 10.3389/fncel.2014.00439 25653587 PMC4299450

[B240] Yu L. M. Y. Goda Y. (2009). Dendritic signalling and homeostatic adaptation. Curr. Opin. Neurobiol. 19, 327–335. 10.1016/j.conb.2009.07.002 19640698

[B241] Yu X. Wang G. Gilmore A. Yee A. X. Li X. Xu T.. (2013). Accelerated experience-dependent pruning of cortical synapses in ephrin-A2 knockout mice. Neuron 80, 64–71. 10.1016/j.neuron.2013.07.014 24094103 PMC3792401

[B242] Yuste R. (2010). Dendritic Spines. Camebridge, MA: The MIT Press.

[B243] Yuste R. Bonhoeffer T. (2001). Morphological changes in dendritic spines associated with long-term synaptic plasticity. Annu. Rev. Neurosci. 24, 1071–1089. 10.1146/annurev.neuro.24.1.1071 11520928

[B244] Zenke F. Hennequin G. Gerstner W. (2013). Synaptic plasticity in neural networks needs homeostasis with a fast rate detector. PLoS Comput. Biol. 9:e1003330. 10.1371/journal.pcbi.1003330 24244138 PMC3828150

[B245] Zhang W. Linden D. J. (2003). The other side of the engram: experience-driven changes in neuronal intrinsic excitability. Nat. Rev. Neurosci. 4, 886–900. 10.1038/nrn1248 14595400

[B246] Zheng P. Dimitrakakis C. Triesch J. (2013). Network self-organization explains the statistics and dynamics of synaptic connection strengths in cortex. PLoS Comput. Biol. 9:e1002848. 10.1371/journal.pcbi.1002848 23300431 PMC3536614

[B247] Zheng P. Triesch J. (2014). Robust development of synfire chains from multiple plasticity mechanisms. Front. Comput. Neurosci. 8:66. 10.3389/fncom.2014.00066 25071537 PMC4074894

[B248] Zhou Q. Homma K. J. Poo M. M. (2004). Shrinkage of dendritic spines associated with long-term depression of hippocampal synapses. Neuron 44, 749–757. 10.1016/j.neuron.2004.11.011 15572107

[B249] Zito K. Scheuss V. Knott G. Hill T. Svoboda K. (2009). Rapid functional maturation of nascent dendritic spines. Neuron 61, 247–258. 10.1016/j.neuron.2008.10.054 19186167 PMC2800307

[B250] Ziv N. E. Smith S. J. (1996). Evidence for a role of dendritic filopodia in synaptogenesis and spine formation. Neuron 17, 91–102. 10.1016/S0896-6273(00)80283-4 8755481

[B251] Zuo Y. Lin A. Chang P. Gan W.-B. (2005a). Development of long-term dendritic spine stability in diverse regions of cerebral cortex. Neuron 46, 181–189. 10.1016/j.neuron.2005.04.001 15848798

[B252] Zuo Y. Yang G. Kwon E. Gan W.-B. (2005b). Long-term sensory deprivation prevents dendritic spine loss in primary somatosensory cortex. Nature 436, 261–265. 10.1038/nature03715 16015331

